# Methylmercury produced in upper oceans accumulates in deep Mariana Trench fauna

**DOI:** 10.1038/s41467-020-17045-3

**Published:** 2020-07-07

**Authors:** Ruoyu Sun, Jingjing Yuan, Jeroen E. Sonke, Yanxu Zhang, Tong Zhang, Wang Zheng, Shun Chen, Mei Meng, Jiubin Chen, Yi Liu, Xiaotong Peng, Congqiang Liu

**Affiliations:** 1 Institute of Surface-Earth System Science, School of Earth System Science, Tianjin University, 300072 Tianjin, China; 2 Laboratoire Géosciences Environnement Toulouse, CNRS/Institute for Research and Development/Université Paul Sabatier–Toulouse III, 31400 Toulouse, France; 3 School of Atmospheric Sciences, Nanjing University, 210023 Jiangsu, China; 4 College of Environmental Science and Engineering, Ministry of Education Key Laboratory of Pollution Processes and Environmental Criteria, Tianjin Key Laboratory of Environmental Remediation and Pollution Control, Nankai University, 38 Tongyan Rd., 300350 Tianjin, China; 5 Deep Sea Science Division, Institute of Deep Sea Science and Engineering, Chinese Academy of Sciences, 572000 Sanya, Hainan China

**Keywords:** Biogeochemistry, Element cycles, Environmental sciences, Marine chemistry

## Abstract

Monomethylmercury (MMHg) is a potent toxin that bioaccumulates and magnifies in marine food webs. Recent studies show abundant methylated Hg in deep oceans (>1000 m), yet its origin remains uncertain. Here we measured Hg isotope compositions in fauna and surface sediments from the Mariana Trench. The trench fauna at 7000–11000 m depth all have substantially positive mass-independent fractionation of odd Hg isotopes (odd-MIF), which can be generated only in the photic zone via MMHg photo-degradation. Given the identical odd-MIF in trench fauna and North Pacific upper ocean (<1000 m) biota MMHg, we suggest that the accumulated Hg in trench fauna originates exclusively from MMHg produced in upper oceans, which penetrates to depth by sorption to sinking particles. Our findings reveal little in-situ MMHg production in deep oceans and imply that anthropogenic Hg released at the Earth’s surface is much more pervasive across deep oceans than was previously thought.

## Abstract (web summary)

Monomethylmercury is a toxin that humans can be exposed to after consumption of seafood in which it has bioaccumulated. Here the authors show that amphipods in the deepest point of the global ocean contain monomethylmercury with surface origins, suggesting rapid sinking of this toxin on particles.

## Introduction

Humans are exposed to toxic monomethylmercury (MMHg) by the consumption of marine fish^1^. Ocean circulation, biogeochemistry, and marine ecology play a crucial role in MMHg exposure to humans. Natural and anthropogenic releases of Hg to air, land, and water eventually enter the oceans by atmospheric deposition and terrestrial discharge^2,3^. Within the oceans, inorganic Hg(II) (IHg(II)) is reduced to gaseous Hg(0) through biotic and abiotic processes, but also transformed to its methylated forms, MMHg and dimethyl-Hg (DMHg), potentially by anaerobic bacteria^4,5^. Particulate organic matter (POM) scavenges IHg(II) in surface oceans and releases it at depth^6,7^. This so-called biological pump results in a macronutrient-like vertical distribution of total Hg concentrations, with low values in epipelagic waters of the surface ocean (0–100 m), increasing through oxygen-depleted mesopelagic waters of the intermediate ocean (100–1000 m), and remaining high in deep ocean waters (>1000 m)^2,8–11^.

MMHg makes up a large fraction of methylated Hg in pelagic marine waters, and its levels and chemical speciation determine the burden available to marine food webs^10–13^. Investigating where and how MMHg is produced in oceans is critically important to understand global Hg cycling and human exposure. At present, MMHg is thought to be produced in situ in the oxygen-depleted zone of mesopelagic waters during microbial remineralization of sinking POM derived from phytoplankton primary production in the photic zone^9,12,14–16^. A fraction of mesopelagic MMHg can be transported to surface oceans via diffusion and upwelling, and in situ MMHg production in oxygenated, epipelagic waters has also been demonstrated^8,10,17–20^. Because MMHg in the epipelagic waters is readily photo-degraded, a depletion of MMHg concentrations is observed in surface oceans^21,22^. Mesopelagic MMHg can penetrate into deep oceans via downwelling or via complexation to or incorporation in sinking POM (i.e., biological pump). However, field data and models indicate that the downward transport of mesopelagic MMHg via downwelling and sinking POM is possibly limited, if not negligible^12,23,24^. Yet, available data, show abundant MMHg and DMHg in deep oceans^8,12,13^, implying that in situ Hg methylation and/or di-methylation in deep oceans is plausible. Due to sparse incubation and measurement of deep waters, the origin (surface or deep), lifetime and fate of deep ocean MMHg and DMHg are therefore largely unknown.

Hg stable isotopes exhibit mass-dependent fractionation (MDF, represented by δ^202^Hg) in all investigated abiotic and biotic Hg transformations, and large mass-independent fractionation of odd-mass number isotopes (odd-MIF, represented by Δ^199^Hg and Δ^201^Hg) in abiotic, photochemical Hg transformations^25,26^. Significant even-mass number MIF (even-MIF, represented by Δ^200^Hg and Δ^204^Hg) has also been observed primarily in atmospheric precipitations, which is proposed to be related to photochemical oxidation of gaseous Hg(0) in the upper atmosphere^27,28^. Due to lack of significant Hg isotope fractionation during accumulation and trophic transfer of MMHg in aquatic food chains^29,30^, Hg isotope compositions of marine biota have demonstrated great potential in tracing the sources, production and degradation of MMHg in oceans^16,31,32^.

**Fig. 1:**
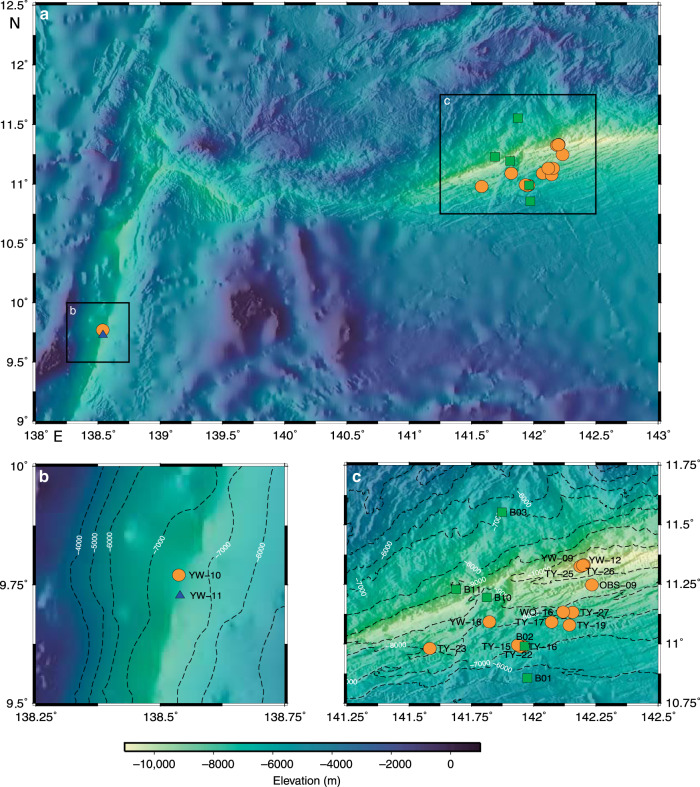
Location map showing the samples from Mariana/Yap trenches. **a** Overview of sampling locations, with the inset **b** for Yap Trench and inset **c** for Mariana Trench. Sediment (green squares), amphipod (orange circles), and snailfish (blue triangle) samples are marked with filled symbols.

The hadal zone of oceans hosts a great diversity of endemic fauna, potentially serving as useful vectors to constrain MMHg dynamics and sources in deep oceans. Here, we measured concentrations and isotope compositions of Hg in endemic amphipods (mostly *Hirondellea gigas*) at water depths of 7000–11,000 m below the surface (mbs) and in benthic sediments at water depths of 5500–9200 mbs from the oligotrophic Mariana Trench in the North Pacific Ocean (NPO, 11.5°N, 142.5°E). Two amphipod samples (*Hirondellea gigas*) and one snailfish sample at water depths of ~8000 mbs from the nearby Yap Trench (9.5°N, 138.5°E) were also collected and measured (Fig. 1 and Supplementary Fig. 1). We find that both MDF and MIF of trench fauna are comparable to those of upper NPO biota MMHg, suggesting that the accumulated Hg in trench fauna originates from MMHg produced in upper oceans.

## Results

### Total Hg and MMHg concentrations

Total Hg concentrations in trench amphipods have an average of 547 ± 230 ng g^−1^ (1 SD, 235 to 1070 ng g^−1^, dry weight, Supplementary Table 1), which is comparable to their counterparts from the abyssal Arctic Ocean (250–1000 ng g^−1^)^33^, but considerably higher than benthic organisms in freshwater and coastal ecosystems (mostly <400 ng g^−1^ on average)^34,35^. The relatively high Hg concentrations in trench amphipods likely relate to their low tissue turnover rates and long life spans^36,37^. Total Hg concentrations of amphipods show no correlation with their depths of occurrence (R^2^ = 0.00, *P* = 0.85), and an insignificantly positive correlation with their body length, a surrogate of life span (R^2^ = 0.12, *P* = 0.08, Supplementary Fig. 2a). MMHg concentrations were measured in nearly half of amphipods, and vary from 8 to 403 ng g^−1^. The fraction of MMHg (MMHg%) is highly variable, from 2 to 59% of total Hg. A significantly negative correlation is observed between MMHg% and body length of amphipods (R^2^ = 0.48, *P* = 0.02, Supplementary Fig. 2b). The snailfish that feeds on amphipods has a total Hg concentration of 970 ng g^−1^ and MMHg concentration of 809 ng g^−1^, which indicates that its MMHg% (83%) is higher than the corresponding values of amphipods (Supplementary Tables 1 and 2).

### Hg isotope compositions

**Fig. 2:**
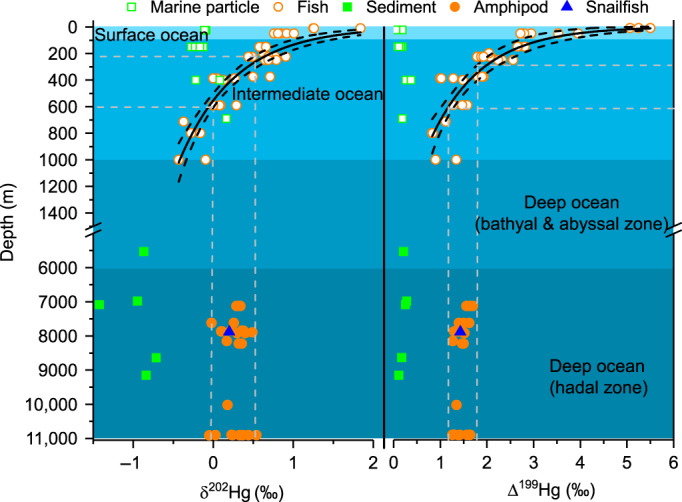
Depth profiles of δ^202^Hg and Δ^199^Hg in trench samples. Samples measured in this study are marked with filled symbols. Δ^201^Hg (not shown) shows similar vertical trend as Δ^199^Hg. Also shown are the marine particles and fishes (open symbols) from North Pacific Subtropical Gyre^16,32^, and the best-fitted regression curves of water depths with δ^202^Hg (R^2^ = 0.812, *p* = 1.11E-16, *n* = 37 samples) and Δ^199^Hg (R^2^ = 0.749, *p* = 1.32E-14, *n* = 37 samples) for fishes. The dashed lines show the water depths predicted by the regression curves using the δ^202^Hg and Δ^199^Hg values of trench fauna. The typical analytic uncertainty of our fauna (sediment) samples was 0.08‰ (0.08‰, two standard deviation) for δ^202^Hg and 0.10‰ (0.04‰, two standard deviation) for Δ^199^Hg.

**Fig. 3:**
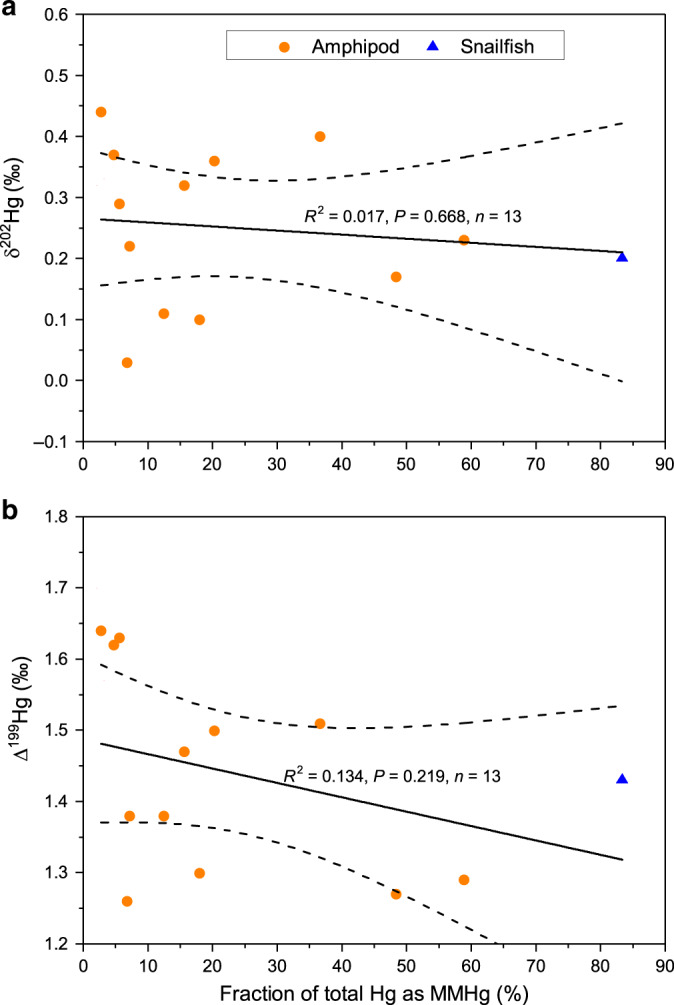
Trench fauna δ^202^Hg, Δ^199^Hg and monomethylmercury fraction. Linear correlations of **a** δ^202^Hg versus monomethylmercury fraction (MMHg%), and **b** of Δ^199^Hg versus monomethylmercury fraction (MMHg%). The typical analytic uncertainty of our fauna samples was 0.08‰ (two standard deviation) for δ^202^Hg and 0.10‰ (two standard deviation) for Δ^199^Hg. n is the number of samples.

The most striking observation of Hg isotopes is that both MDF (δ^202^Hg: −0.05 to 0.54‰, *n* = 28) and odd-MIF (Δ^199^Hg: 1.26 to 1.70‰, Δ^201^Hg: 1.01 to 1.37‰, *n* = 28) of trench amphipods are comparable to those of NPO fishes at 300–600 mbs^16,32^, despite their dramatic difference in depths (Fig. 2). The variability of MDF and odd-MIF for amphipods is only ~0.5‰, and their variations are not correlated with MMHg% (2 to 59%, Fig. 3), depths of occurrence (7000–11,000 mbs), sampling locations (Yap and Mariana trenches) or sampling time (July 2016 to March 2017) (Supplementary Fig. 3 and Supplementary Table 2). However, positive correlations are seen between δ^202^Hg and Hg concentrations (235–1070 ng g^−1^) (R^2^ = 0.27, *P* = 0.01), and between Δ^199^Hg and body length (1–4.5 cm) (R^2^ = 0.18, *P* = 0.04) (Supplementary Fig. 3). Although these positive correlations suggest that physiological processes in amphipods may affect their Hg isotope compositions, they can only explain 20–30% of Hg isotope variations. Hg isotope compositions of the snailfish are within those of amphipods (Fig. 2). In contrast, the trench sediments have negative δ^202^Hg (−0.96 ± 0.27‰, 1 SD, *n* = 5) and very small, positive odd-MIF (Δ^199^Hg = 0.20 ± 0.07‰, Δ^201^Hg = 0.18 ± 0.04‰, 1 SD, *n* = 5) (Supplementary Table 3). The even-MIF of trench fauna (amphipod and snailfish) is characterized by positive Δ^200^Hg (0.02–0.13‰) and negative Δ^204^Hg (−0.18 to −0.03‰), which is very similar to those of trench sediments, and upper marine particles and fishes from the upper NPO^16,32^ (Supplementary Fig. 4).

## Discussion

**Fig. 4:**
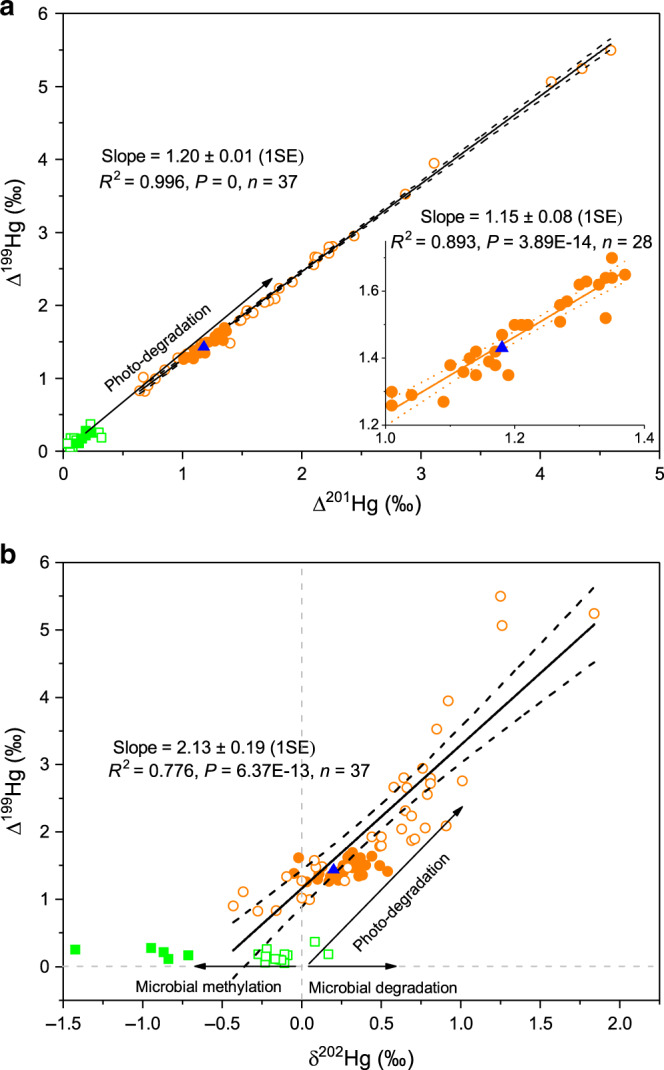
Correlations of mercury isotope values in trench samples. **a** Δ^201^ Hg versus Δ^199^Hg, and **b** δ^202^Hg versus Δ^199^Hg. Also shown are the marine particles and fishes from North Pacific Subtropical Gyre^16,32^, and the linear regression lines of Hg isotope compositions for fishes^16,32^. The inset in **a** shows the linear regression line of Δ^201^ Hg versus Δ^199^Hg in trench fauna. Symbols for samples are the same as in Fig. 2. SE is the standard error, and *n* is the number of samples. The arrows show the experimental isotope fractionation trajectories during microbial methylation of IHg(II)^51–53^, and microbial and photodegradation of MMHg^39,42^. The typical 2 SD analytic uncertainty of our fauna (sediment) samples was 0.08‰ (0.08‰, two standard deviation) for δ^202^Hg, and 0.10‰ (0.04‰, two standard deviation) for Δ^199^Hg and Δ^201^Hg.

The similar MIF signatures in deep fauna and surface fishes reveal important insight into hadal Hg provenance. First, Δ^200^Hg has thus far been used as a conservative tracer at the Earth’s surface, and is thought to be generated during net Hg(0) oxidation in the upper atmosphere^28,38^. The identical, positive Δ^200^Hg in deep and surface fauna (Supplementary Fig. 4) suggests that the IHg(II) precursor to MMHg in the deep and surface fauna is similar and likely of atmospheric Hg origin. Second, odd-MIF mainly accompanies photoreduction of IHg(II) and photodegradation of MMHg in aqueous environments^39,40^. The Δ^199^Hg/Δ^201^Hg ratio is a diagnostic of photochemical reaction types, with an experimental value of ~1.3 for MMHg photodegradation and ~1.0 for IHg(II) photoreduction^39^. The deep fauna all have positive odd-MIF values, and Δ^199^Hg and Δ^201^Hg are highly correlated along a linear slope of 1.15 ± 0.08 (1SE, Fig. 4a). This Δ^199^Hg/Δ^201^Hg slope is comparable to slopes (~1.2) reported for fishes from upper NPO and other coastal and open marine waters^16,31,32,41^, suggesting that the odd-MIF in the trench fauna is produced by similar surface ocean MMHg photodegradation processes. Experimental photodegradation of aqueous MMHg has also been suggested to induce a characteristic Δ^199^Hg/δ^202^Hg ratio of 2.43 ± 0.10^39^. A similar slope is observed in NPO fishes (2.13 ± 0.19, 1SE, Fig. 4b)^16,32^. Most of our trench fauna samples lie on the NPO fish slope, but do not show sufficient variation in both Δ^199^Hg and δ^202^Hg to confirm the Δ^199^Hg/δ^202^Hg slope as a diagnostic. Minor δ^202^Hg deviations in some fauna samples (Fig. 4b) are likely caused by small amounts of non-photochemical, microbial degradation of MMHg, which results in no MIF but increases the δ^202^Hg of residual MMHg accumulated in fauna^42^.

The isotope compositions of total Hg in trench amphipods are representative of the isotope signatures of MMHg in the same amphipods, despite the relatively low MMHg% of 2–59%. The reason for this is that most of the IHg(II) in the amphipods is likely produced by in vivo degradation of MMHg rather than assimilation of IHg(II) from the ambient environments. This is supported by multiple lines of evidence. (1) The significantly negative correlation between MMHg% and body length of amphipods (Supplementary Fig. 2b) indicates that in vivo demethylation of MMHg likely occurs in the amphipods. MMHg demethylation is commonly observed in organisms with a gastro-intestinal tract including isopods, fish and mammals^43–45^. (2) The trench fauna MIF values exhibit a Δ^199^Hg/Δ^201^Hg regression slope of 1.15 that is diagnostic of MMHg photodegradation rather than IHg(II) photoreduction. This suggests that most of the IHg(II) fraction in amphipods inherits its Δ^199^Hg and Δ^201^Hg signatures from ingested MMHg, following in vivo demethylation. (3) Trench amphipods have very consistent, high δ^202^Hg (0.27 ± 0.14‰, 1 SD) and Δ^199^Hg (1.47 ± 0.13‰, 1 SD), and exhibit little variation in both signatures in spite of large variations in MMHg% (Fig. 3). The snailfish with 83% of MMHg% has nearly the same Hg isotope composition as trench amphipods which have varying and low MMHg%. If significant fraction of IHg(II) was to be assimilated from the surrounding environments, we would expect a more scattered Hg isotope distribution due to the mixing of IHg(II) and MMHg. We would expect that the trench fauna with high IHg(II)% to have low δ^202^Hg and Δ^199^Hg, because IHg(II) in the oceans has relatively low δ^202^Hg and Δ^199^Hg, as seen from our measured seafloor sediments and previously reported open ocean particles^32^ and sediments^46,47^, and coastal seawater^48,49^. (4) Insignificant differences (<0.1‰) in Hg isotope compositions are observed between the whole tissues and the separated tissues (muscle, lipid, and gut contents) of amphipods, although their Hg concentrations and speciation vary significantly (Supplementary Table 2). This suggests that the internal distribution or in vivo transformation of Hg species do not significantly fractionate Hg isotopes between MMHg and IHg(II) in amphipods. In the following we discuss the origin of fauna MMHg, based on all available open ocean Hg isotope data, and make a number of deductions that constrain the deep ocean MMHg cycling.

IHg(II) in sinking marine particles of different sizes and depths in the NPO has a constant Δ^199^Hg of 0.16 ± 0.09‰ (1 SD)^32^, which is found back at depth in Mariana sediments (0.20 ± 0.07‰, 1 SD) (Fig. 2). Potential MMHg produced in situ in trench sediments or deep water column should therefore inherit the low Δ^199^Hg of ~0.2‰. MMHg produced from hydrothermal vent fluids is expected to have near-zero Δ^199^Hg, because Hg of geological origin generally has near-zero MIF^50^. The trench amphipods are benthic scavengers, which mainly reside in the top of the sediments surrounding the hydrothermal vent fields, and feed on sinking particles. If MMHg is produced in the sediments, diffusing to the water column, or in hydrothermal fluids or in the deep water column, it would transfer the near-zero to very small Δ^199^Hg to the amphipods. The occurrence of significant, 1.47 ± 0.13‰ (1 SD), Δ^199^Hg in the deepest marine fauna on Earth suggests that this Δ^199^Hg is not produced in situ in the deep ocean environments. Instead, elevated MMHg Δ^199^Hg can only be acquired during photodegradation of MMHg in the euphotic zone (Fig. 2), since potential microbial methylation of IHg(II) and non-photodegradation of MMHg in deep waters would not induce MIF^42,51–53^. We therefore suggest that the trench MMHg is produced in the surface ocean and subsequently transport to depth before entering the deep ocean food web.

**Fig. 5:**
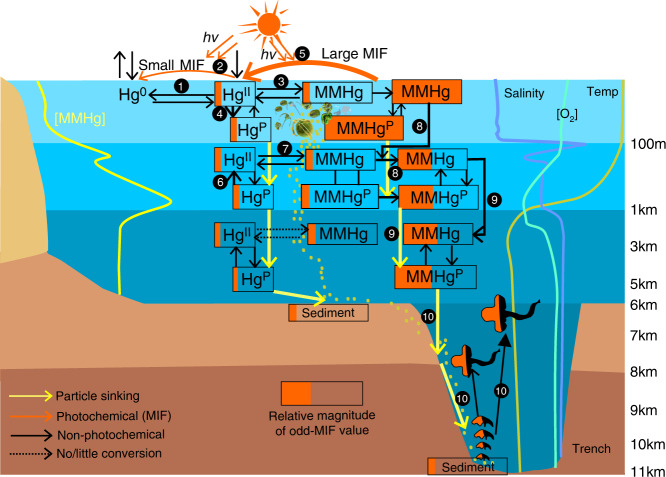
Conceptual illustration of mercury cycling in trench systems. Atmospheric inorganic Hg^II^ (IHg(II)) is deposited to the epipelagic waters where it is microbially (1) or photochemically (2) reduced to Hg(0), microbially methylated to monomethylmercury (MMHg) (3), and scavenged as Hg^P^ by sinking particles (4). Photo-reduction of IHg(II) results in residual IHg(II) with small odd-MIF (2), while photodegradation of MMHg results in residual MMHg with large odd-MIF (5). The sinking particles are decomposed and release IHg(II) in the mesopelagic waters (6) where microbial methylation produces MMHg that inherits the small odd-MIF of IHg(II) (7). The mixing of MMHg produced in epipelagic and mesopelagic waters in the intermediate ocean (8) results in MMHg with moderate odd-MIF^16^, which is then transported to the deep ocean by sinking particles (9). Since no/little MMHg is produced in the deep ocean, the MMHg with moderate odd-MIF is incorporated into deep food webs (10). Also shown are typical depth profiles of NPO dissolved MMHg concentrations^10,12^, Mariana dissolved oxygen [O_2_]^59^, and Mariana seawater salinity and temperature measured in this study. The hadal food web is based on Gerringer^37^.

IHg(II) and MMHg can be transported from surface to intermediate and even deep oceans by sinking particles, and by subducting water masses during deep water formation^7,54^. The latter transport pathway is unlikely in the Mariana/Yap trench system because there is little deep water formation in the NPO^55^. The lifetime of MMHg with a surface ocean Δ^199^Hg of ~1.5‰ would have to be thousands of years for the MMHg to arrive in the Mariana Trench by diffusion and advection, which is also unlikely^4^. The mixing of MMHg produced in epipelagic and mesopelagic waters has been previously proposed to account for the observed declines of δ^202^Hg and Δ^199^Hg values in NPO fishes with their feeding depths (Fig. 2)^16^. We observe that Δ^199^Hg values in trench fauna at 7000–11000 mbs are comparable to those of NPO fishes at 300–600 mbs (Fig. 2). We use mean NPO surface and intermediate dissolved MMHg concentrations to estimate a weighted average Δ^199^Hg of 1.44 ± 0.75‰ (1 SD) in surface/intermediate food web MMHg (see Methods). The close correspondence of the weighted mean Δ^199^Hg of NPO surface/intermediate MMHg to trench fauna Δ^199^Hg (1.47 ± 0.13‰, 1 SD) suggests that MMHg is transported to depth on sinking particles. The importance of particles to move IHg(II) from the upper ocean to greater depths have been recently highlighted^6,7^. A study on amphipods from the Mariana Trench and two other Pacific trenches revealed that the bomb ^14^C signals of the surface ocean could rapidly penetrate into the deepest ocean via fast sinking of surface POM^56^. It is thus likely that, similar to IHg(II), the MMHg produced in the surface and intermediate waters above the trenches transfers to the deep ocean through sinking of surface POM as well. A conceptual model of Hg cycling in the Mariana/Yap trench system is shown in Fig. 5, illustrating the methylation and degradation of Hg, and how the surface MMHg odd-MIF propagates to deep food webs.

The δ^202^Hg values of trench fauna at 7000–11000 mbs (0.27 ± 0.14‰, 1 SD) are ~0.3‰ higher than the weighted mean δ^202^Hg (−0.05 ± 0.30‰, 1 SD) of NPO surface/intermediate MMHg (see Methods). This magnitude of the δ^202^Hg shift is comparable to the ~0.4‰ shift observed during microbial degradation of MMHg in culture, which increases the δ^202^Hg value of residual MMHg^42^. Thus, microbial degradation of MMHg likely occur during MMHg transport from the upper ocean to depth. The ensuing question is why MMHg is degraded, but not produced in the deep ocean that occupies more than 70% of the whole Ocean’s volume. Both non-photochemical degradation and production of MMHg are predominantly mediated by microbes. The microbes that produce MMHg are thought to be mostly anaerobic bacteria with *hgcAB* genes, while the microbes that degrade MMHg could be either aerobic or anaerobic bacteria^57,58^. The microbes that are capable of MMHg degradation are thus more widespread in nature, including oceans. The deep marine waters are cold, nutrient-limited, and oxygen-rich^59^ (Fig. 5), which might inhibit the methylation ability of anaerobic bacteria^60^. The bacteria in deep oceans that are capable to demethylate MMHg also likely exist in the guts of trench amphipods^44,45^, and cause the in vivo demethylation of MMHg we infer above.

The Hg isotope compositions of trench fauna clearly indicate that bioavailable MMHg in the planet’s deepest ocean is produced in the upper ocean, and that consequently little MMHg is produced in the deep ocean. We use a binary mixing model based on Δ^199^Hg to estimate that 37–48% and 52–63% of MMHg in the trench fauna is derived from the surface and the intermediate ocean, respectively (see Methods). Several centuries of human activities have severely perturbed natural Hg cycling, elevating atmospheric Hg levels at the Earth’s surface by a factor of 3–7^2,61^. Currently, ~300 Mmol of anthropogenic Hg is estimated to reside in oceans, with two thirds in the upper ocean and the remainder subducting in North Atlantic Deep Water and Antarctic Bottom Water^2^. The tight MMHg linkage between trench fauna and the upper ocean suggest that anthropogenic Hg is much more pervasive across the world’s oceans than was previously thought, and may reach the deepest ocean by fast particle sinking. Ongoing surface ocean warming was recently suggested to increase MMHg levels in the upper ocean^62^, which would likely rapidly propagate to the hadal ecosystems.

Our findings not only inform on the source of MMHg in the deep ocean, but also allow us to speculate about the production of DMHg in the deep ocean. Mean oceanic dissolved MMHg and DMHg profiles show similar concentration trends at depth throughout the world’s oceans^10–13,24^. The limited global variation in MMHg and DMHg concentrations indicates slow transformations, supported by our finding that little IHg(II) methylation takes place in the deep NPO. The exact link between MMHg and DMHg in marine waters is presently unclear. State of the art marine Hg models exclude direct DMHg formation from IHg(II)^24,63^, supported by slow observed rates^17^, but include microbial methylation of IHg(II) to form MMHg, and subsequent microbial methylation of MMHg to form DMHg in deep waters. Here, we find that deep ocean MMHg is supplied from the upper ocean by particle sinking rather than in situ microbial methylation. DMHg is a dissolved gas and is unlikely to be transported on particles. If in situ microbial MMHg production in the deep waters is limited, then microbial methylation of MMHg to form DMHg is likely also limited. This leads us to speculate that deep ocean DMHg is abiotically produced, possibly on particle surfaces as supported by recent laboratory experiments^64^.

## Methods

### Sampling

Endemic amphipods were captured by bait traps installed on the deep-sea lander vehicles (@Tianya, @Yuanwei, @Wanquan) that were operated by the Chinese Academy of Sciences during several cruises between July 2016 and March 2017. The lander vehicles were deployed to the seafloor of Mariana (11.5°N, 142.5°E) and Yap trenches (9.5°N, 138.5°E) in the NPO at depths of 7000–11,000 mbs for 12–24 h and monitored by cameras (Fig. 1 and Supplementary Table 1). The bait was enclosed in an isolated mesh bag of a funnel trap, which allowed for odor plume diffusion and prevented consumption of the bait. The captured amphipods were frozen immediately at −80 °C upon loading on deck. The amphipods mainly comprised of *Hirondellea gigas* with body lengths of 1–4.5 cm except one amphipod *Alicella gigantea* with a body length of 12 cm (Supplementary Fig. 1). After returning to laboratory, amphipods were rinsed with Milli-Q water before tissue separation, freeze-drying and homogenization. The amphipods mostly had empty gut contents, and were processed as whole tissues after removing the heads to avoid potential contamination by bait. To increase the sampling representativeness, several amphipod individuals of similar sizes were usually combined to one sample. Three large-size amphipod samples were separated for muscle, lipid and gut contents using pre-cleaned surgical scissors and tweezers to assess whether internal distribution or in vivo transformation of Hg species induce Hg isotope fractionation (Supplementary Table 2). We also trapped one snailfish that primarily fed on amphipods in the Yap Trench, for which we separated its muscle and processed it in the same way as amphipods. Ocean floor sediments at depths of 5500–9200 mbs were collected with a box corer that was launched by a geological winch during one cruise operated by the Chinese Academy of Sciences in 2016. Immediately after recovery, short sediment cores were collected by pushing PVC tubes (L = 80 cm, ID = 9 cm) into the sediment blocks. The top 0–6 cm layers of sediments from all the short cores were then subsampled and stored at −20 °C before freeze-drying and homogenization.

### Total Hg and MMHg concentration measurement

Total Hg concentrations in the homogenized freeze-dried samples were measured by a Lumex RA-915F Mercury Analyzer. The samples were combusted in the PYRO-915+ unit, and the evaporated Hg(0) was then purified and transferred to an analytical cell before measurement by differential Zeeman atomic absorption spectrometry (AAS). Total Hg concentrations in the digested sample solutions used for Hg isotope ratio analysis were measured by a Tekran 2600 cold vapor atomic fluorescence spectrometer (CV-AFS) according to US-EPA method 1631E. MMHg was determined in amphipod and snailfish samples by a Tekran 2700 gas chromatograph-CV-AFS after solvent extraction using KOH/CH_3_OH solution, and ethylation by NaBEt_4_ in closed purge vessels^35^. The combined 2 SD analytic uncertainties of total Hg concentrations, as evaluated by periodically measured certified reference materials (CRMs, GBW07405, soil; DORM-4, dogfish muscle), were <5% for both Lumex RA-915F and Tekran 2600. The combined 2 SD analytic uncertainties for MMHg concentrations of DORM-4, were <15%. The relative standard deviations of sample/CRM duplicates were <5% for THg and <8% for MMHg concentrations.

### Hg isotope ratio measurement

According to the measured total Hg concentrations in freeze-dried samples, ~0.2–0.6 g of each fauna sample was digested using 4 mL double-distilled HNO_3_ and 4 mL super-pure H_2_O_2_ in a 30 mL Teflon lined vessel heated by a programmed microwave digestion system. Sediments were pre-concentrated for Hg using the combustion-trapping method^65^, and 10 mL of 40% double-distilled acid (2HNO_3_/1HCl, v/v) was used to trap the volatilized Hg(0). Procedural blanks and CRMs were processed with the samples in a same manner. The procedural blanks accounted for <1% of Hg mass in the samples, and the Hg digestion and preconcentration recoveries were in the range of 88–110% for both the samples and procedural CRMs. The processed sample solutions were diluted with Milli-Q water to Hg concentrations of 0.5–2 ng g^−1^, and were measured for Hg isotope ratios by coupling a customized cold vapor generation system to multi-collector inductively coupled plasma mass spectrometry (MC-ICPMS, Nu Plasma 3D at Tianjin University, China). The typical sensitivity for ^202^Hg was ~2 V per ng g^−1^ Hg at a solution uptake rate of 0.8 mL min^−1^. Instrumental mass bias was corrected by both an internal NIST 997 Tl standard solution (supplied via Aridus II desolvation nebulizer system) using the exponential fractionation law and NIST 3133 Hg standard-sample bracketing method. The bracketed NIST 3133 solutions were matched to the sample solutions within 5% in both acid matrix and Hg concentrations. The Faraday cups were positioned to simultaneously collect all seven Hg isotopes and two Tl isotopes. Acquisition time was 7 min (5 blocks, 20 cycles, 4.2 s of integration time) with 3 min of initial uptake time. Between samples, the system was washed with the sample matrix solution for 7 min to ensure that the blank signals were <1% of the preceding sample or standard signals.

Hg isotope ratio is expressed as δ^xxx^Hg (‰, xxx = 199, 200, 201, 202, 204) by normalizing to a common NIST 3133 Hg standard:

1 $$\delta^{xxx} {\mathrm{Hg}}\left( {‰} \right) = \left[ \left( {\!\,}^{xxx}{\mathrm{Hg}}/{\,\!}^{198}{\mathrm{Hg}} \right)_{{\mathrm{sample}}}/\left( {\,\!}^{xxx}{\mathrm{Hg}}/{\,\!}^{198}{\mathrm{Hg}} \right)_{{\mathrm{NIST}}\,{\mathrm{3133}}} - 1 \right] \times 1000$$

MIF value is denoted as Δ^*xxx*^Hg (‰, *xxx* = 199, 200, 201, 204), representing the difference between the measured δ^*xxx*^Hg value and that predicted from δ^202^Hg using a kinetic MDF law^66^:

2 $$\Delta ^{xxx}{\mathrm{Hg}}\left( {‰} \right) = {\updelta}^{xxx}{\mathrm{Hg}} - ^{xxx}{\beta} \times {\updelta}^{202}{\mathrm{Hg}}$$

The mass-dependent scaling factor ^*xxx*^*β* is 0.2520 for ^199^Hg, 0.5024 for ^200^Hg, 0.7520 for ^201^Hg, and 1.4930 for ^204^Hg.

Hg isotope ratios of secondary standard NIST 3177 solution and procedural CRMs (GBW07310, DORM-4) analyzed during different analytic sessions (Supplementary Table 4) were in agreement with those reported in previous studies^35,65,66^. The typical 2 SD analytic uncertainties of samples were estimated as the larger 2 SD uncertainties of Hg isotope ratios in NIST 3177 and GBW07310 (sediment) or DORM-4 (amphipod and snaifish). The 2 SD uncertainties of Hg isotope ratios in samples with replicate analyses were applied as the analytic uncertainties only when they were larger than the typical 2 SD analytic uncertainties.

### Estimate of weighted MMHg isotope values of upper NPO

The weighted MMHg isotope values (δ^202^Hg_upper_, Δ^199^Hg_upper_, Δ^201^Hg_upper_) of the upper NPO (0–1000 mbs) are estimated by the following binary mixing equations using a Monte Carlo simulation approach (*n* = 10,000 times) through the pseudorandom number generation function of the MatLab software (R2016b, MathWorks):

3 $${\updelta}^{{\mathrm{202}}}{\mathrm{Hg}}_{{\mathrm{upper}}} = ({\mathrm{C}}_{{\mathrm{surf}}} \times {\updelta}^{{\mathrm{202}}}{\mathrm{Hg}}_{{\mathrm{surf}}} + {\mathrm{C}}_{{\mathop{\rm{int}}} } \times {\updelta}^{{\mathrm{202}}}{\mathrm{Hg}}_{{\mathop{\rm{int}}} })/({\mathrm{C}}_{{\mathrm{surf}}} + {\mathrm{C}}_{{\mathop{\rm{int}}} })$$

4 $$\Delta ^{{\mathrm{199}}}{\mathrm{Hg}}_{{\mathrm{upper}}} = ({\mathrm{C}}_{{\mathrm{surf}}} \times \Delta ^{{\mathrm{199}}}{\mathrm{Hg}}_{{\mathrm{surf}}} + {\mathrm{C}}_{{\mathop{\rm{int}}} } \times \Delta ^{{\mathrm{199}}}{\mathrm{Hg}}_{{\mathop{\rm{int}}} })/({\mathrm{C}}_{{\mathrm{surf}}} + {\mathrm{C}}_{{\mathop{\rm{int}}} })$$

5 $$\Delta ^{{\mathrm{201}}}{\mathrm{Hg}}_{{\mathrm{upper}}} = ({\mathrm{C}}_{{\mathrm{surf}}} \times \Delta ^{{\mathrm{201}}}{\mathrm{Hg}}_{{\mathrm{surf}}} + {\mathrm{C}}_{{\mathop{\rm{int}}} } \times \Delta ^{{\mathrm{201}}}{\mathrm{Hg}}_{{\mathop{\rm{int}}} })/({\mathrm{C}}_{{\mathrm{surf}}} + {\mathrm{C}}_{{\mathop{\rm{int}}} })$$

In which, δ^202^Hg_surf_, Δ^199^Hg_surf_ and Δ^201^Hg_surf_ of MMHg in the surface ocean (after photo-demethylation) are represented by those of NPO fishes at 0–100 mbs (δ^202^Hg = 1.06 ± 0.35‰, Δ^199^Hg = 3.84 ± 1.15‰, Δ^201^Hg = 3.13 ± 0.97‰, 1 SD)^16,32^, and δ^202^Hg_int_, Δ^199^Hg_int_ and Δ^201^Hg_int_ of MMHg in the intermediate ocean (without photo-demethylation) are represented by those of NPO marine particles (mainly IHg(II)) after correcting for −0.5‰ shift of δ^202^Hg during microbial methylation of IHg(II)^16,32,51^ (δ^202^Hg = −0.62 ± 0.13‰, Δ^199^Hg = 0.16‰ ± 0.09‰, Δ^201^Hg = 0.13‰ ± 0.10‰, 1 SD). C_surf_ and C_int_ are represented by mean NPO surface (18 ± 7 pM, 1 SD) and intermediate (38 ± 19 pM, 1 SD) MMHg concentrations, respectively (see Supplementary Note 1). The calculated upper NPO MMHg has a weighted mean of −0.05 ± 0.30‰ (1 SD) for δ^202^Hg, 1.44 ± 0.75‰ (1 SD) for Δ^199^Hg and 1.16 ± 0.60‰ (1 SD) for Δ^201^Hg.

### Estimate of MMHg contributions in trench fauna

Since total Hg in the trench fauna is derived from MMHg in the upper ocean, MMHg contributions (f_surf_: fraction from the surface ocean; f_int_: fraction from the intermediate ocean) to the trench fauna can be estimated by the following binary mixing model using a Monte Carlo simulation approach (*n* = 10,000 times) through the pseudorandom number generation function of the MatLab software (R2016b, MathWorks):

6 $$\Delta ^{{\mathrm{199}}}{\mathrm{Hg}}_{{\mathrm{sam}}} = {\mathrm{f}}_{{\mathrm{surf}}} \times \Delta ^{{\mathrm{199}}}{\mathrm{Hg}}_{s{\mathrm{urf}}} + {\mathrm{f}}_{{\mathop{\rm{int}}} } \times \Delta ^{{\mathrm{199}}}{\mathrm{Hg}}_{{\mathop{\rm{int}}} }$$

7 $${\mathrm{f}}_{s{\mathrm{urf}}} + {\mathrm{f}}_{{\mathop{\rm{int}}} }{\mathrm{ = }}1$$

where Δ^199^Hg_sam_, Δ^199^Hg_surf_, and Δ^199^Hg_int_ represent Δ^199^Hg values of trench fauna (measured), MMHg produced in the surface ocean (3.84 ± 1.15‰, 1 SD) and MMHg produced in the intermediate ocean (0.16‰ ± 0.09‰), respectively^16,32^.

### Description of statistical analysis

All the statistical analyses were performed using OriginPro 9 except for mixing models by MatLab R2016b. Nonlinear exponential fitting was applied for Fig. 2 and linear fitting for other figures. The fitting curves are bounded by 95% confidence bands, and Pearson’s R-Square and *P*-values are calculated by algorithms of the software. Two-side analysis of variance (ANOVA) was used to asses if the regression slope is significantly different from zero at the 0.05 level.

### Reporting summary

Further information on research design is available in the Nature Research Reporting Summary linked to this article.

## Supplementary information

Supplementary Information

Peer Review File

Reporting Summary

## Data Availability

The authors declare that the main data supporting the findings of this study are available within the article and its Supplementary Information file. Extra data are available from the corresponding author upon request.

## Code availability

Binary mixing model codes are available at the following link: [https://github.com/ruoyusun/Mixing-models].
