# Supplementary material for: Methylmercury produced in upper oceans accumulates in deep Mariana Trench fauna
Source: Nat Commun. 2020 Jul 7;11:3389. doi: 10.1038/s41467-020-17045-3 (PMC7341844; doi:10.1038/s41467-020-17045-3)
Supplement: Supplementary file 1 — Supplementary Information [file 41467_2020_17045_MOESM1_ESM.pdf]

Supplementary Information for

# Methylmercury produced in upper oceans accumulates in deep Mariana Trench fauna

by Sun et al.

## Supplementary Note

### Supplementary Note1: Total and methylated mercury depth profiles

Total and methylated Hg (monomethylmercury-MMHg, dimethylmercury-DMHg) depth profiles in the marine water column have been recently reported for the North Pacific Ocean for several cruises (*1-4*). Supplementary Fig. 5 shows the reported depth profiles of THg and methylated Hg in the tropical water column from western (11-22°N, 129-148°E, near the studied Mariana/Yap trenches) (*2*) and eastern North Pacific Ocean (12-23°N, 152-155°W, near Hawaii where fish and particle Hg isotope data are available) (*1, 3*). Although differing in THg and methylated Hg levels, their depth profiles are broadly similar: enrichment in the sub-thermocline waters and depletion in the surface waters.

MMHg and DMHg were only separately quantified for the single SAFe station (30°N, 140°W) in eastern North Pacific (*4*), and for four stations (4-17°N, 154-157°W) in the tropical North Pacific Ocean (*3*). After removing the data points of MMHg concentrations equal to and/or below the MMHg detection limit, we calculated the mean surface (18 ± 7 pM, 1SD) and intermediate (38 ± 19 pM, 1SD) MMHg concentrations in the North Pacific Ocean.

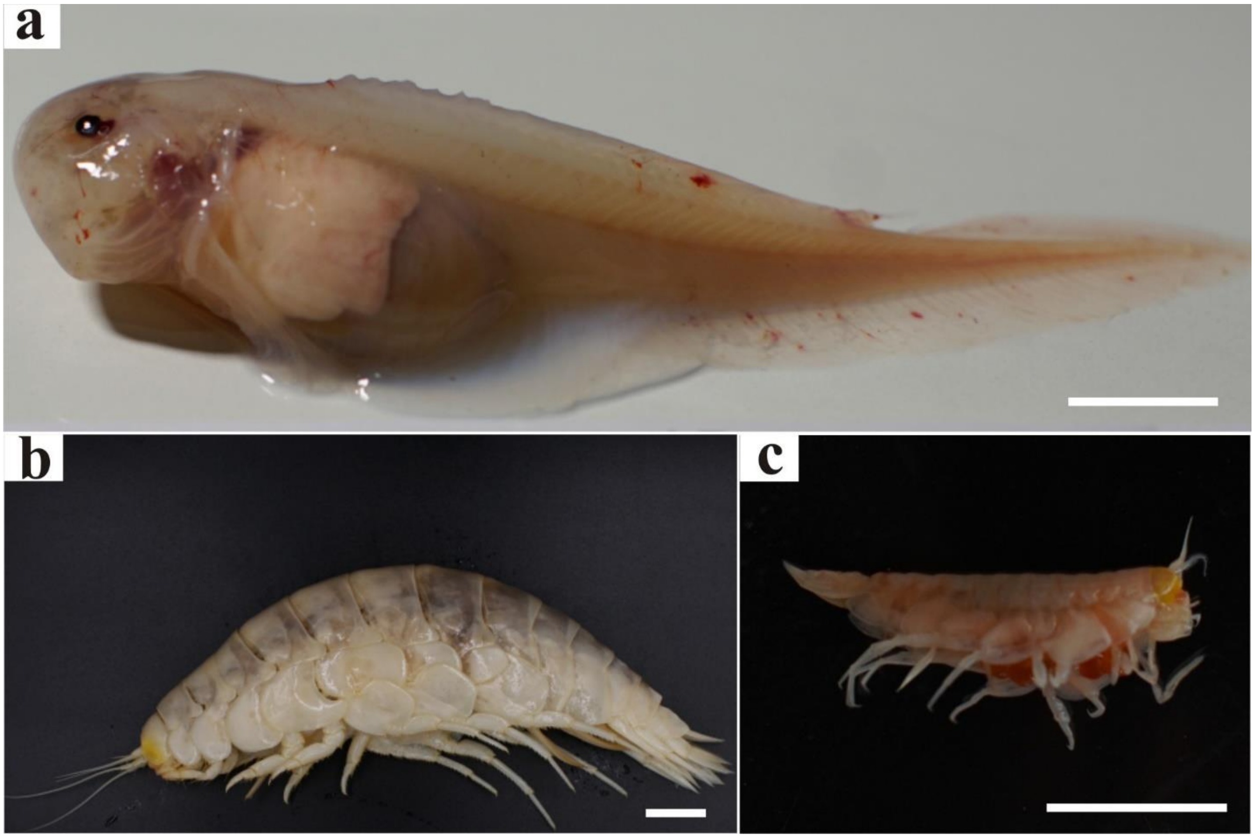


**Supplementary Figure 1. Typical specimens of Mariana/Yap trench fauna.** Snailfish (**a**) and two amphipod species (**b**: Alicella gigantean; **c**: Hirondellea gigas). Scale bar = 20 mm.

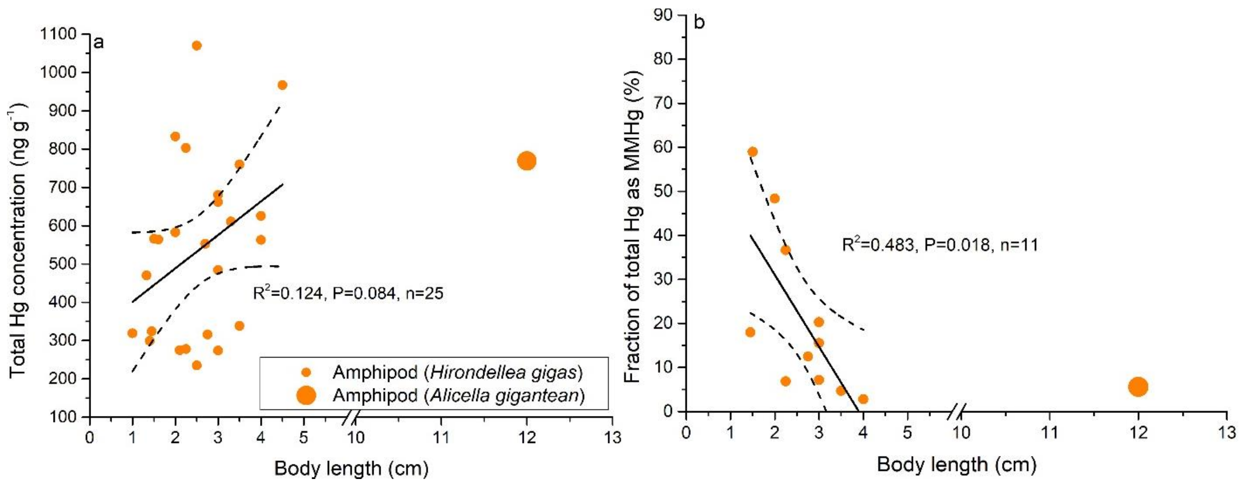


**Supplementary Figure 2. Body length, mercury concentration and methylmercury fraction.** Linear correlations of (**a**) body lengths versus total Hg concentrations, and (**b**) of body lengths versus monomethylmercury fractions (MMHg%) in small-size (1-4.5 cm) amphipods (*Hirondellea gigas*). Note that one large-size (12 cm) amphipod of a different species (*Alicella gigantean*, 12 cm) is not included in the correlation analysis. n is the number of samples.

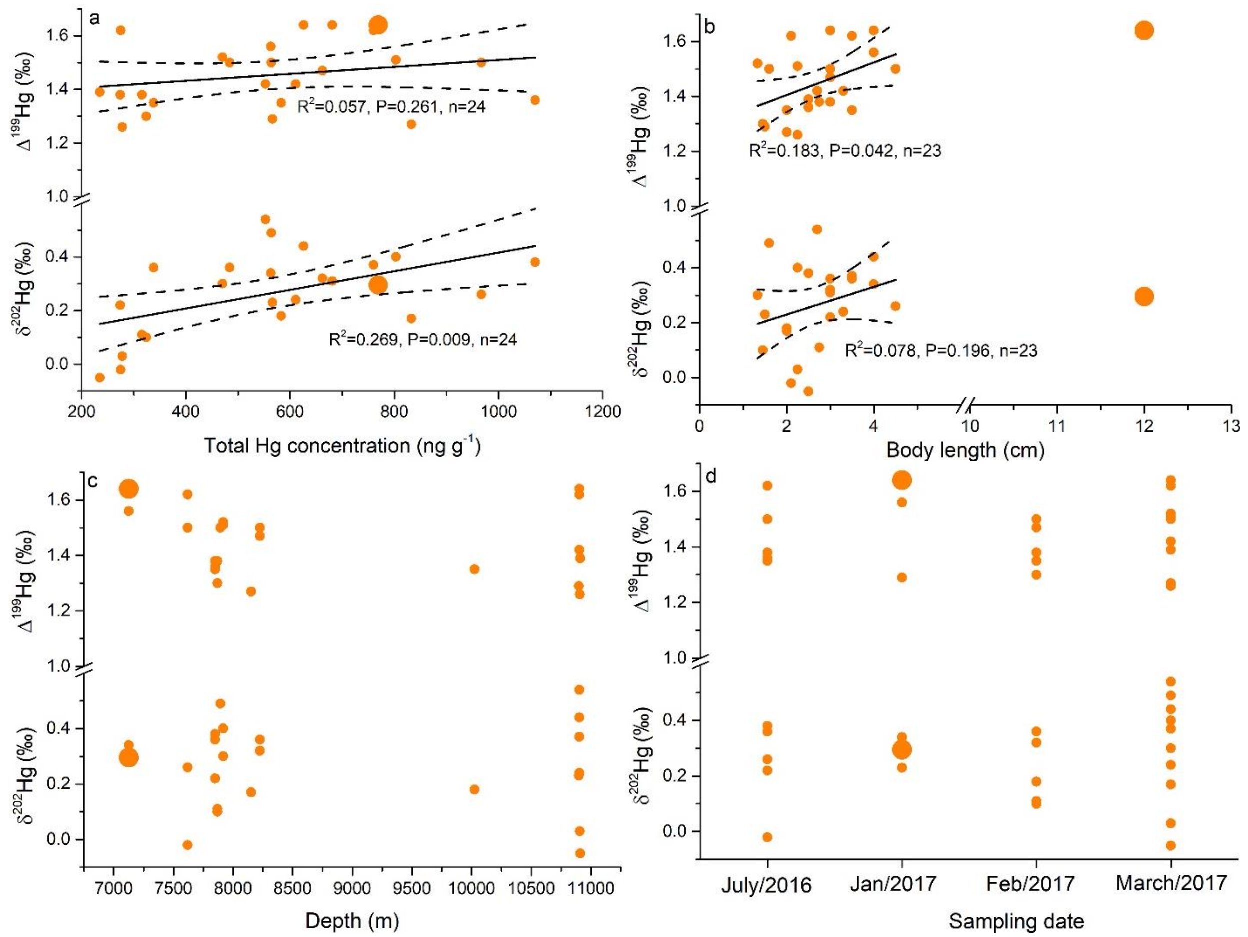


**Supplementary Figure 3. Plots of mercury isotope values with other parameters.** Linear correlations of (**a**) $\delta^{202}$Hg or $\Delta^{199}$Hg versus total Hg concentrations, and (**b**) of $\delta^{202}$Hg or $\Delta^{199}$Hg versus body lengths. No correlations are observed for (**c**) $\delta^{202}$Hg or $\Delta^{199}$Hg versus depths of occurrence, and for (**d**) $\delta^{202}$Hg or $\Delta^{199}$Hg versus sampling dates. Symbols for samples are the same as in Supplementary Figure 2. Note that one large-size (12 cm) amphipod of a different species (*Alicella gigantean*, 12 cm) is not included in the correlation analysis of (**b**). The typical 2SD (standard deviation) analytic uncertainty of our fauna samples was 0.08‰ for $\delta^{202}$Hg and 0.10‰ for $\Delta^{199}$Hg. n is the number of samples.

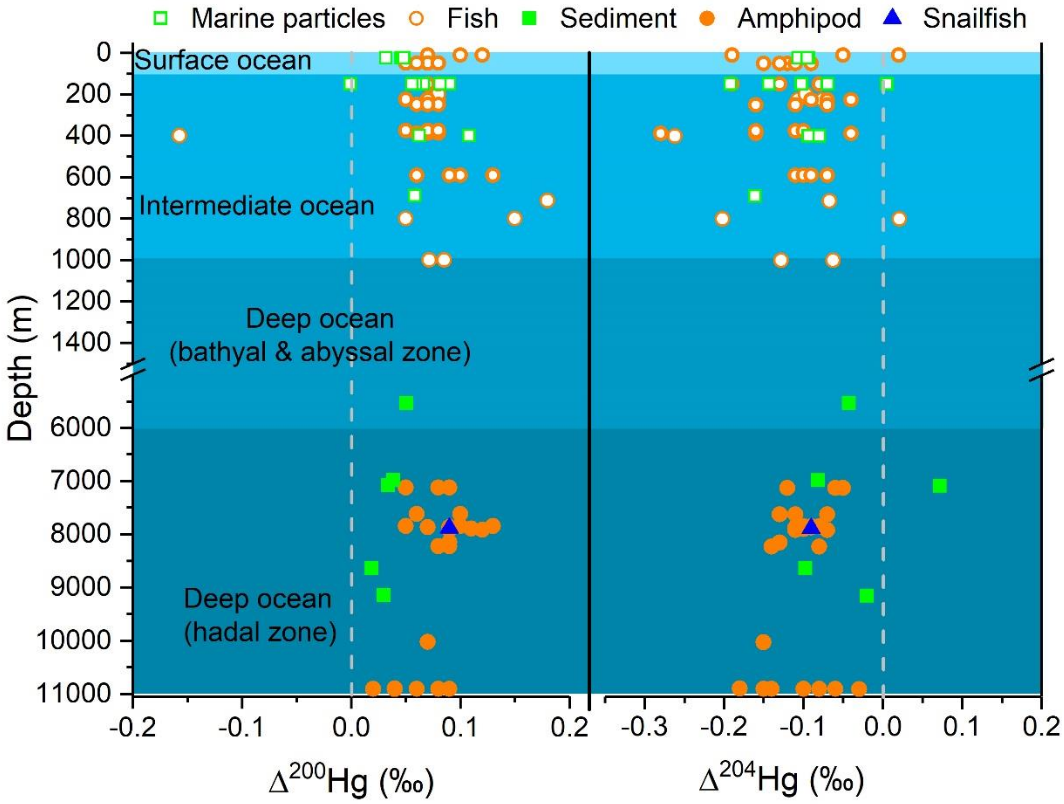


**Supplementary Figure 4. Depth profiles of $\Delta^{200}$Hg and $\Delta^{204}$Hg of in trench samples.** Sediment (green squares), amphipod (orange circles) and snailfish (blue triangle) samples are marked with filled symbols. Also shown are the marine particles and fishes (open symbols) from the North Pacific Subtropical Gyre (*5*, *6*). The typical 2SD (standard deviation) analytic uncertainty of our fauna (sediment) samples was 0.04‰ (0.06‰) for $\Delta^{200}$Hg and 0.08‰ (0.08‰) for $\Delta^{204}$Hg.

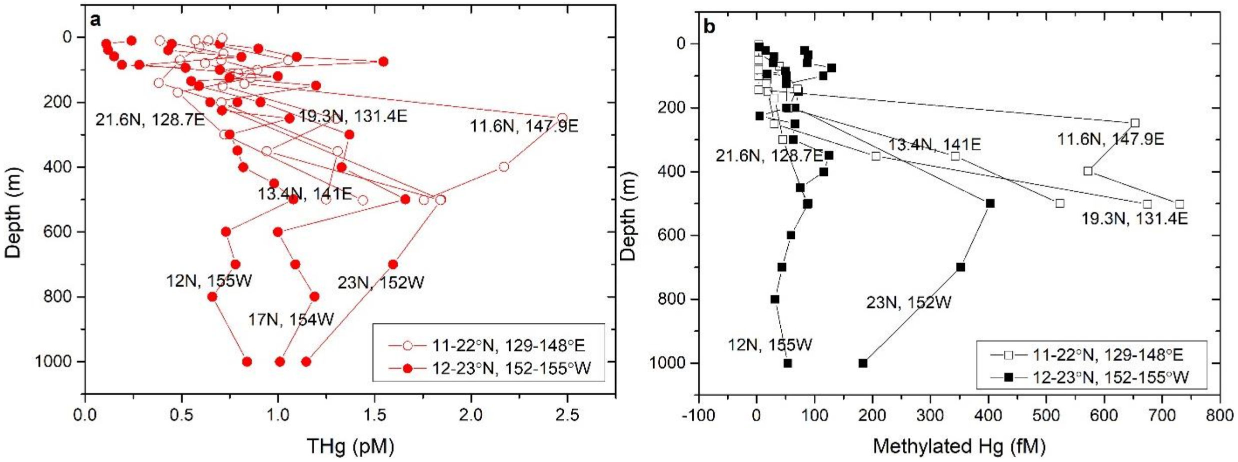


**Supplementary Figure 5. Depth profiles of mercury in the tropical water masses.** (**a**) Total Hg concentration and (**b**) methylated Hg concentrations in the upper ocean from eastern (*2*) and western North Pacific Ocean (*1*, *3*).

**Supplementary Table 1. Sampling information of trench fauna and their total Hg and MMHg concentrations of whole body tissues**

| Sample ID | Lander ID | Species | Collection date (yyyymmdd) | Longitude | Latitude | Collection depth (m) | Number of Individuals per sample | Mean body length (cm) | Total Hg concentration (ng $g^{-1}$, dry weight) | MMHg concentration (ng $g^{-1}$, dry weight) |
|---|---|---|---|---|---|---|---|---|---|---|
| TY-15 | Tian Ya | *Hirondellea gigas* | 20160715 | 141°57.51′E | 10°59.35′N | 6985 | 3 | 1.0 | 319 | NA |
| TY-16 | Tian Ya | *Hirondellea gigas* | 20160719 | 141°56.87′E | 10°59.25′N | 7034 | 2 | 1.4 | 299 | NA |
| TY-17-1 | Tian Ya | *Hirondellea gigas* | 20160722 | 142°04.38′E | 11°05.46′N | 7850 | 1 | 3.5 | 338 | NA |
| TY-17-2 | Tian Ya | *Hirondellea gigas* | 20160722 | 142°04.38′E | 11°05.46′N | 7850 | 1 | 2.5 | 1070 | NA |
| TY-17-3 | Tian Ya | *Hirondellea gigas* | 20160722 | 142°04.38′E | 11°05.46′N | 7850 | 1 | 3.0 | 274 | 19.7 |
| TY-19-1 | Tian Ya | *Hirondellea gigas* | 20160726 | 142°08.65'E | 11°04.71'N | 7619 | 1 | 4.5 | 967 | NA |
| TY-19-2 | Tian Ya | *Hirondellea gigas* | 20160726 | 142°08.65'E | 11°04.71'N | 7619 | 2 | 2.1 | 275 | NA |
| TY-22-1 | Tian Ya | *Hirondellea gigas* | 20170130 | 141°56.19'E | 10°59.67'N | 7125 | 1 | 4.0 | 563 | NA |
| TY-22-2 | Tian Ya | *Hirondellea gigas* | 20170130 | 141°56.19'E | 10°59.67'N | 7125 | 1 | 3.0 | 681 | NA |
| TY-22-3 | Tian Ya | *Alicella gigantea* | 20170130 | 141°56.19'E | 10°59.67'N | 7125 | 1 | 12 | 769 | 42.8 |
| TY-23-1 | Tian Ya | *Hirondellea gigas* | 20170210 | 141°35.02'E | 10°58.84'N | 8226 | 1 | 3.0 | 484 | 98.3 |
| TY-23-2 | Tian Ya | *Hirondellea gigas* | 20170210 | 141°35.02'E | 10°58.84'N | 8226 | 1 | 3.0 | 662 | 103.0 |
| TY-25 | Tian Ya | *Hirondellea gigas* | 20170305 | 142°11.42'E | 11°19.50'N | 10908 | 2 | 2.3 | 278 | 19.0 |
| TY-26 | Tian Ya | *Hirondellea gigas* | 20170308 | 142°11.32′E | 11°19.50′N | 10911 | 1 | 2.5 | 235 | NA |
| TY-27 | Tian Ya | *Hirondellea gigas* | 20170311 | 142°09.42′E | 11°07.95′N | 8152 | 5 | 2.0 | 833 | 403 |
| YW-9 | Yuan Wei | *Hirondellea gigas* | 20170128 | 142°11.96'E | 11°19.79'N | 10901 | 4 | 1.5 | 566 | 333.6 |
| YW-12-1 | Yuan Wei | *Hirondellea gigas* | 20170302 | 142°12.11'E | 11°19.92'N | 10904 | 1 | 3.3 | 611 | NA |
| YW-12-2 | Yuan Wei | *Hirondellea gigas* | 20170302 | 142°12.11'E | 11°19.92'N | 10904 | 1 | 2.7 | 553 | NA |
| YW-12-3 | Yuan Wei | *Hirondellea gigas* | 20170302 | 142°12.11'E | 11°19.92'N | 10904 | 1 | 3.5 | 760 | 35.5 |
| YW-12-4 | Yuan Wei | *Hirondellea gigas* | 20170302 | 142°12.11'E | 11°19.92'N | 10904 | 1 | 4.0 | 626 | 17.6 |
| YW-16-1 | Yuan Wei | *Hirondellea gigas* | 20170313 | 141°49.34′E | 11°5.51′N | 7895 | 5 | 1.6 | 564 | NA |
| WQ-16-1 | Wan Quan | *Hirondellea gigas* | 20170312 | 142°07.13'E | 11°08.05'N | 7917 | 2 | 2.3 | 803 | 294.2 |

| | | | | | | | | | | |
|---|---|---|---|---|---|---|---|---|---|---|
| WQ-16-2 | Wan Quan | *Hirondellea gigas* | 20170312 | 142°07.13'E | 11°08.05'N | 7917 | 3 | 1.3 | 470 | NA |
| OBS-09 | OBS | *Hirondellea gigas* | 20170226 | 142°14.04'E | 11°14.86'N | 10026 | 1 | 2.0 | 583 | NA |
| YW-10-1 | Yuan Wei | *Hirondellea gigas* | 20170219 | 138°32.24'E | 9°46.24'N | 7869 | 2 | 2.8 | 316 | 39.4 |
| YW-10-2 | Yuan Wei | *Hirondellea gigas* | 20170219 | 138°32.24'E | 9°46.24'N | 7869 | 4 | 1.5 | 324 | 58.2 |
| YW-11 | Yuan Wei | *snailfish* | 20170223 | 138°32.38′E | 9°43.66′N | 7884 | 1 | 25 | 970 | 808.5 |

Note all samples were collected in the Mariana Trench except for YW-10-1, -10-2 and -11 from the Yap Trench. NA: not available.

**Supplementary Table 2. Total Hg and MMHg concentrations, and Hg isotope compositions of different body tissues of trench fauna**

| Sample ID | Tissue type | Total Hg concentration (ng $g^{-1}$, dry weight) | MMHg concentration (ng $g^{-1}$, dry weight) | n | $\delta^{202}$Hg (‰) | 2SD (‰) | $\Delta^{199}$Hg (‰) | 2SD (‰) | $\Delta^{200}$Hg (‰) | 2SD (‰) | $\Delta^{201}$Hg (‰) | 2SD (‰) | $\Delta^{204}$Hg (‰) | 2SD (‰) |
|---|---|---|---|---|---|---|---|---|---|---|---|---|---|---|
| TY-15 | Whole body | 319 | NA | | NA | | NA | | NA | | NA | | NA | |
| TY-16 | Whole body | 299 | NA | | NA | | NA | | NA | | NA | | NA | |
| TY-17-1 | Whole body | 338 | NA | 2 | 0.36 | 0.15 | 1.35 | 0.13 | 0.10 | 0.02 | 1.19 | 0.03 | -0.09 | 0.06 |
| TY-17-2 | Whole body | 1070 | NA | 1 | 0.38 | | 1.36 | | 0.05 | | 1.12 | | -0.08 | |
| TY-17-3 | Whole body | 274 | 19.7 | 1 | 0.22 | | 1.38 | | 0.13 | | 1.17 | | -0.10 | |
| TY19-1 | Whole body | 967 | NA | 2 | 0.26 | 0.04 | 1.50 | 0.03 | 0.06 | 0.02 | 1.20 | 0.05 | -0.11 | 0.03 |
| | Muscle | 403 | NA | 1 | 0.26 | | 1.40 | | 0.10 | | 1.13 | | -0.07 | |
| TY19-2 | Whole body | 275 | NA | 2 | -0.02 | 0.04 | 1.62 | 0.04 | 0.10 | 0.01 | 1.33 | 0.01 | -0.13 | 0.02 |
| TY-22-1 | Whole body | 563 | NA | 1 | 0.34 | | 1.56 | | 0.09 | | 1.27 | | -0.06 | |
| TY-22-2 | Whole body | 681 | NA | 1 | 0.31 | | 1.64 | | 0.08 | | 1.35 | | -0.12 | |
| TY-22-3 | Whole body w/o gut | 769 | 42.8 | 1 | 0.29 | | 1.63 | | 0.05 | | 1.31 | | -0.12 | |
| | Duplicate | | | 1 | 0.30 | | 1.65 | | 0.08 | | 1.37 | | -0.05 | |
| | Gut | 580 | 12.0 | 1 | 0.32 | | 1.70 | | 0.09 | | 1.35 | | -0.06 | |
| TY-23-1 | Whole body | 484 | 98.3 | 1 | 0.36 | | 1.50 | | 0.08 | | 1.21 | | -0.14 | |
| TY-23-2 | Whole body | 662 | 103.0 | 1 | 0.32 | | 1.47 | | 0.09 | | 1.18 | | -0.08 | |
| TY-25 | Whole body | 278 | 19.0 | 1 | 0.03 | | 1.26 | | 0.06 | | 1.01 | | -0.15 | |
| TY-26 | Whole body | 235 | NA | 1 | -0.05 | | 1.39 | | 0.04 | | 1.16 | | -0.03 | |
| TY-27 | Whole body | 833 | 403 | 1 | 0.17 | | 1.27 | | 0.09 | | 1.09 | | -0.13 | |
| YW-9 | Whole body | 566 | 333.6 | 1 | 0.23 | | 1.29 | | 0.04 | | 1.04 | | -0.18 | |
| YW-12-1 | Whole body | 611 | NA | 1 | 0.24 | | 1.42 | | 0.08 | | 1.17 | | -0.08 | |
| YW-12-2 | Whole body | 553 | NA | 1 | 0.54 | | 1.42 | | 0.08 | | 1.14 | | -0.06 | |
| YW-12-3 | Whole body | 760 | 35.5 | 1 | 0.37 | | 1.62 | | 0.02 | | 1.30 | | -0.10 | |
| YW-12-4 | Whole body | 626 | 17.6 | 2 | 0.44 | 0.06 | 1.64 | 0.01 | 0.09 | 0.02 | 1.34 | 0.13 | -0.14 | 0.08 |

| | | | | | | | | | | | | | | | |
|---|---|---|---|---|---|---|---|---|---|---|---|---|---|---|---|
| | Lipid | 246 | 7.9 | 1 | 0.33 | | 1.57 | | 0.02 | | 1.28 | | -0.10 | |
| YW-16-1 | Whole body | 564 | NA | 1 | 0.49 | | 1.50 | | 0.11 | | 1.22 | | -0.10 | |
| WQ-16-1 | Whole body | 803 | 294.2 | 1 | 0.40 | | 1.51 | | 0.09 | | 1.27 | | -0.11 | |
| WQ-16-2 | Whole body | 470 | NA | 2 | 0.30 | 0.03 | 1.52 | 0.01 | 0.12 | 0.03 | 1.34 | 0.11 | -0.07 | 0.06 |
| OBS-09 | Whole body | 583 | NA | 1 | 0.18 | | 1.35 | | 0.07 | | 1.14 | | -0.15 | |
| YW-10-1 | Whole body | 316 | 39.4 | 1 | 0.11 | | 1.38 | | 0.07 | | 1.10 | | -0.09 | |
| YW-10-2 | Whole body | 324 | 58.2 | 1 | 0.10 | | 1.30 | | 0.09 | | 1.01 | | -0.11 | |
| YW-11 | Muscle | 970 | 808.5 | 1 | 0.20 | | 1.43 | | 0.09 | | 1.18 | | -0.09 | |

All samples were collected in the Mariana Trench except for YW-10-1, -10-2 and -11 from the Yap Trench. n: number of measurement replicates; NA: not available.

**Supplementary Table 3. Total Hg concentrations and Hg isotope compositions of sediments**

| Sample ID | Longitude | Latitude | Collection depth (m) | Total Hg concentrations (ng $g^{-1}$) | $\delta^{202}Hg$ (‰) | $\Delta^{199}Hg$ (‰) | $\Delta^{200}Hg$ (‰) | $\Delta^{201}Hg$ (‰) | $\Delta^{204}Hg$ (‰) |
|---|---|---|---|---|---|---|---|---|---|
| B01 | 141° 58.50'E | 10° 51.36'N | 5525 | 12.1 | -0.87 | 0.21 | 0.05 | 0.19 | -0.04 |
| B02 | 141° 57.87'E | 10° 59.38'N | 6980 | 6.0 | -0.95 | 0.28 | 0.04 | 0.19 | -0.12 |
| B03 | 141° 52.38'E | 11° 33.19'N | 7082 | 11.9 | -1.42 | 0.25 | 0.03 | 0.24 | 0.07 |
| B10 | 141° 48.70'E | 11° 11.70'N | 8638 | 21.9 | -0.71 | 0.17 | 0.02 | 0.15 | -0.10 |
| B11 | 141° 41.38'E | 11° 13.71'N | 9150 | 6.6 | -0.84 | 0.11 | 0.03 | 0.13 | -0.02 |

**Supplementary Table 4. Summary of Hg isotope compositions and uncertainties in certified reference materials**

| | n | $\delta^{202}$Hg (‰) | 2SD (‰) | $\Delta^{199}$Hg (‰) | 2SD (‰) | $\Delta^{200}$Hg (‰) | 2SD (‰) | $\Delta^{201}$Hg (‰) | 2SD (‰) | $\Delta^{204}$Hg (‰) | 2SD (‰) |
|---|---|---|---|---|---|---|---|---|---|---|---|
| **NIST 3177** | 45 | -0.51 | 0.08 | 0.00 | 0.04 | 0.01 | 0.04 | -0.02 | 0.04 | -0.02 | 0.08 |
| **DORM-4** | 13 | 0.43 | 0.06 | 1.70 | 0.10 | 0.06 | 0.04 | 1.38 | 0.10 | -0.07 | 0.06 |
| **GBW07310** | 4 | -0.49 | 0.06 | -0.27 | 0.02 | -0.01 | 0.06 | -0.24 | 0.04 | -0.01 | 0.05 |

n: number of measurement replicates

## Supplementary References


1. E. M. Sunderland, D. P. Krabbenhoft, J. W. Moreau, S. A. Strode, W. M. Landing, Mercury sources, distribution, and bioavailability in the North Pacific Ocean: Insights from data and models. *Global Biogeochem. Cycles* **23**, GB2010 (2009).
2. H. Kim *et al.*, Methylmercury mass budgets and distribution characteristics in the western Pacific Ocean. *Environ. Sci. Technol.* **51**, 1186-1194 (2017).
3. K. M. Munson, C. H. Lamborg, G. J. Swarr, M. A. Saito, Mercury species concentrations and fluxes in the Central Tropical Pacific Ocean. *Global Biogeochem. Cycles* **29**, 656-676 (2015).
4. C. R. Hammerschmidt, K. L. Bowman, Vertical methylmercury distribution in the subtropical North Pacific Ocean. *Mar. Chem.* **132-133**, 77-82 (2012).
5. J. D. Blum, B. N. Popp, J. C. Drazen, C. Anela Choy, M. W. Johnson, Methylmercury production below the mixed layer in the North Pacific Ocean. *Nat. Geosci.* **6**, 879-884 (2013).
6. L. C. Motta *et al.*, Mercury cycling in the North Pacific Subtropical Gyre as revealed by mercury stable isotope ratios. *Global Biogeochem. Cycles* **33**, 777-794 (2019).
