# Supplementary material for: Methylmercury produced in upper oceans accumulates in deep Mariana Trench fauna
Source: Nat Commun. 2020 Jul 7;11:3389. doi: 10.1038/s41467-020-17045-3 (PMC7341844; doi:10.1038/s41467-020-17045-3)
Supplement: Supplementary file 3 — Reporting Summary [file 41467_2020_17045_MOESM3_ESM.pdf]

nature research

Corresponding author(s): Yi Liu, Xiaotong Peng

Last updated by author(s): Jun 5, 2020

Reporting Summary

Nature Research wishes to improve the reproducibility of the work that we publish. This form provides structure for consistency and transparency in reporting. For further information on Nature Research policies, see Authors & Referees and the Editorial Policy Checklist.

Statistics

For all statistical analyses, confirm that the following items are present in the figure legend, table legend, main text, or Methods section.

n/a	Confirmed	
☐	☒	The exact sample size (*n*) for each experimental group/condition, given as a discrete number and unit of measurement
☐	☒	A statement on whether measurements were taken from distinct samples or whether the same sample was measured repeatedly
☐	☒	The statistical test(s) used AND whether they are one- or two-sided<br>*Only common tests should be described solely by name; describe more complex techniques in the Methods section.*
☒	☐	A description of all covariates tested
☐	☒	A description of any assumptions or corrections, such as tests of normality and adjustment for multiple comparisons
☐	☒	A full description of the statistical parameters including central tendency (e.g. means) or other basic estimates (e.g. regression coefficient) AND variation (e.g. standard deviation) or associated estimates of uncertainty (e.g. confidence intervals)
☐	☒	For null hypothesis testing, the test statistic (e.g. F, t, r) with confidence intervals, effect sizes, degrees of freedom and P value noted<br>*Give P values as exact values whenever suitable.*
☒	☐	For Bayesian analysis, information on the choice of priors and Markov chain Monte Carlo settings
☒	☐	For hierarchical and complex designs, identification of the appropriate level for tests and full reporting of outcomes
☐	☒	Estimates of effect sizes (e.g. Cohen's *d*, Pearson's *r*), indicating how they were calculated

Our web collection on statistics for biologists contains articles on many of the points above.

Software and code

Policy information about availability of computer code

Data collection	All the data and algorithms are provided in the Method section and Supporting Information
Data analysis	We use a Monte Carlo simulation in MatLab software (R2016b, MathWorks) to perform the binary mixing models. The MatLab codes are deposited in GitHub [https://github.com/ruoyusun/Mixing-models]. We use OriginPro 9 software for the statistical analyses.

For manuscripts utilizing custom algorithms or software that are central to the research but not yet described in published literature, software must be made available to editors/reviewers. We strongly encourage code deposition in a community repository (e.g. GitHub). See the Nature Research guidelines for submitting code & software for further information.

Data

Policy information about availability of data

All manuscripts must include a data availability statement. This statement should provide the following information, where applicable:

- Accession codes, unique identifiers, or web links for publicly available datasets
- A list of figures that have associated raw data
- A description of any restrictions on data availability

All data are available in the Supplementary Information

Field-specific reporting

Please select the one below that is the best fit for your research. If you are not sure, read the appropriate sections before making your selection.

☐ Life sciences ☐ Behavioural & social sciences ☒ Ecological, evolutionary & environmental sciences

Ecological, evolutionary & environmental sciences study design

All studies must disclose on these points even when the disclosure is negative.

Study description	This study measures the concentrations and isotope compositions of mercury in fauna collected from ocean trenches, and aims to investigate the origin and cycling of methylmercury in the deep ocean. The data are measured by following strict laboratory protocols, and the experimental QA/QC are provided in the method section of main text. In summary, 26 amphipods and 1 snailfish were measured for total mercury concentrations with relative standard deviations of replicates (n = 27) <5%, and 12 amphipods and 1 snailfish were measured for methylmercury concentrations with relative standard deviations of replicates (n = 13) <8%. Mercury isotope compositions were measured on 24 amphipods and 1 snailfish, with a good reproducibility for replicates (n = 5).
Research sample	The samples used are amphipod and snailfish collected from the deep ocean trenches. These organisms are abundant in the trenches, were selected to represent the deep-sea fauna. A full description of these samples including their species, lengths and living depths are provided in the method section of main text and Supporting Information.
Sampling strategy	The samples are collected by deep-sea lander vehicles on the seafloor of Mariana and Yap trenches. The sizes of samples used in this study depend on their availability and the representativeness of measurement results.
Data collection	Data were collected from the instruments (AFS, AAS, and MC-ICPMS) by following strict protocols as described in the method section of main text, and were recorded by J. Y., M. M. and R. S.
Timing and spatial scale	Depending on the cruise plans and landing conditions, the samples were collected between July 2016 and March 2017, and covered the Mariana (11.5°N, 142.5°E) and Yap trenches (9.5°N, 138.5°E), among the deepest locations of the Earth.
Data exclusions	No data are excluded during the analysis
Reproducibility	We used standard reference materials and sample replicates (n = 27 for total mercury test, n = 13 for methylmercury test, and n = 5 for mercury isotope test) to verify the reproducibility of measurements, and all results are reproducible within uncertainty.
Randomization	To increase the sampling representativeness, several sample individuals of similar sizes were combined into one sample. No randomization is needed.
Blinding	Blinding is not relevant to this study, and we only need to measure the concentrations and isotope compositions of mercury in samples

Did the study involve field work? ☒ Yes ☐ No

Field work, collection and transport

Field conditions	The samples are collected by deep-sea lander vehicles on the seafloor of Mariana and Yap trenches. The sampling locations have water depths between 6000-10000 m with high pressure (~1000 times of normal air pressure) and low temperature (2-3 Celsius degree).
Location	The sampling locations are at the seafloor of Mariana (11.5°N, 142.5°E) and Yap trenches (9.5°N, 138.5°E), western North Pacific Ocean. Endemic amphipods and snailfish were collected at 7000-11000 m, and sediments were collected at 5500-9200 m.
Access and import/export	The samples are collected from international waters (Mariana/Yap trenches) by the Institute of Deep Sea Science and Engineering, Chinese Academy of Sciences, with permit by the Federated States of Micronesia between July 2016 and March 2017.
Disturbance	We used bait traps to capture the fauna, and box corer to sample the sediments in the deep sea environments, thus the disturbance is very limited.

Reporting for specific materials, systems and methods

We require information from authors about some types of materials, experimental systems and methods used in many studies. Here, indicate whether each material, system or method listed is relevant to your study. If you are not sure if a list item applies to your research, read the appropriate section before selecting a response.

Materials & experimental systems

n/a	Involved in the study	
☒	☐	Antibodies
☒	☐	Eukaryotic cell lines
☒	☐	Palaeontology
☐	☒	Animals and other organisms
☒	☐	Human research participants
☒	☐	Clinical data

Methods

n/a	Involved in the study	
☒	☐	ChIP-seq
☒	☐	Flow cytometry
☒	☐	MRI-based neuroimaging

Animals and other organisms

Policy information about studies involving animals; ARRIVE guidelines recommended for reporting animal research

Laboratory animals	This study does not involve laboratory animals
Wild animals	The used amphipods and snailfish live in the deep-sea environments, and are not protected by relevant laws. These organisms were caught by deep-sea lander vehicles and transported to laboratory by the scientific research vessel@Tanshuo. After the study, the captive animals were kept at a −80 °C freezer.
Field-collected samples	These field samples were frozen immediately at −80 °C upon loading on deck of research vessel
Ethics oversight	No ethical approval or guidance was required for the studied organisms

Note that full information on the approval of the study protocol must also be provided in the manuscript.
