# Supplementary material for: Methylmercury produced in upper oceans accumulates in deep Mariana Trench fauna
Source: Nat Commun. 2020 Jul 7;11:3389. doi: 10.1038/s41467-020-17045-3 (PMC7341844; doi:10.1038/s41467-020-17045-3)
Supplement: Supplementary file 2 — Peer Review File [file 41467_2020_17045_MOESM2_ESM.docx]

Peer Review Information

**Journal:** Nature Communications

**Manuscript title:** Methylmercury produced in upper oceans accumulates in deep Mariana Trench fauna

**Corresponding author name(s):** Prof Yi Liu

**Editorial notes:**

**none**

**Reviewer comments & decisions:**

**Reviewer comments, first version:**

Reviewer #1 (Remarks to the Author):

Methylated Hg in the deep ocean, especially DMHg, has been given very little attention by the oceanographic community, with no study reporting beyond concentration values. This study provides a novel investigation of the source of MMHg to deep-sea biota, while providing new insight on the source of DMHg to the deep ocean water column. The results of this study show that MMHg produced in the surface ocean from anthropogenic Hg affects not only epipelagic and mesopelagic organisms, but deep-sea ecosystems as well. This study is also of high value to the modeling community, as increased efforts and data are needed to improve models of methylated Hg in the global ocean. A few minor edits are suggested below.

Line 45: This statement needs to be supported by a reference. A lot of studies in the Pacific report equal amounts of MMHg and DMHg in the water column.
Line 67: The authors should elaborate on “skeptically related” if this statement is to remain in the manuscript.
Line 225: There is a new paper on Hg cycling genes in the ocean that supports the discussion here (Bowman et al. 2019; Limnology & Oceanography). The authors found no evidence of hgcAB in marine waters, but did find merB in the deep ocean.


Reviewer #2 (Remarks to the Author):

The Sun et al. study uses stable isotope signatures of Hg in North Pacific deep trench amphipods and marine sediment in comparison to previously published data from surface and intermediate ocean depths to assess MMHg cycling and sources in deep water. The study identifies sinking particles formed in the upper ocean as the primary source of MMHg in the deep ocean, rather than in situ production, and uses Monte Carlo simulations to determine the overall sources MMHg in deep trench fauna from surface and intermediate sources.

The application of isotope signatures to deep sea organisms seems a natural progression based on the last decade or so of marine Hg literature. The emphasis on sinking particles as the Hg source to deep sea amphipods does not appear novel. Why the authors assume that sediments would be a potential food source for freely moving amphipods captured from bait traps is unclear to me. Evidence that these amphipods can feed from sediment would support the authors' claims that Hg from surface particles are more bioavailable than Hg in sediments.

The primary conclusions of the study are based on analysis of THg and MMHg (typically % THg has MMHg) from collected fauna and marine sediments. My concerns over the conclusions drawn from the provided data include:
1. The statistical analysis presented in Fig S2B, Fig S4A, and Fig S4B are inconsistent with the data provided in Table S1 or Table S2. The length relationship displayed in Fig S2B cannot be based on 25 individual measurements, since only 11 amphipod samples were analyzed for MMHg. Likewise, the sample size given in Fig S4 is only possible if both the TY-22-3 duplicate and gut sample and the YW-12-4 lipid sample are counted as individual samples, which is an inaccurate description, as they represent either a measure of error (duplicate) or subsets of the wholebody samples (gut/lipid). The authors need to verify their correlation calculations in order to correct their figures and the resulting interpretation in L95-100 and L114-L119.
2. The overall percent THg as MMHg in the measured samples (2-59 %) is low compared to fish. The mean THg as MMHg value is ~20 %, whereas the comparison to published fish data, in which MMHg often exceeds 90 % of the measured THg. Despite the fact that the majority of Hg in all but 1 sample (YW-9) is present as THg, the authors interpret the stable isotopic signatures as representing sources of MMHg, rather than THg. Thus, the focus on MMHg is poorly supported by the data, specifically in the following sections:
a. L134-135: The source allocation for deep ocean MMHg sources rather than THg.
b. L172-176: The discussion of trench MMHg rather than trench THg.
c. L189-191: The supposition that MMHg is transported to depth, rather than THg.
d. L193-195: The assessment of MMHg, rather than THg, sinking rates.
e. L117-125: Why MMHg degredation is invoked when the majority of the Hg isotopes represent those of THg.
f. L139-152: Any discussion of DMHg production at depth is dependent on the
3. The sediment samples are bulk samples from box cores, with no information provided concerning the sediment horizon captured (these mixed sediments may be far from newly deposited sediment from falling particles). The authors compare these directly to the collection of suspended sediment samples collected from in situ pumps in intermediate waters, which by definition collect primarily neutrally buoyant, rather than sinking particles. Furthermore, only THg analysis was performed on the sediment samples and if the percent THg as MMHg in these sediments is lower than zooplankton, often < 1 % in marine sediments, the isotopic signatures of the sediment poorly represent those of MMHg.

A thorough critique of the Monte Carlo simulations falls outside my field of expertise. However, from reviewing the code, it appeared to me that the percentage THg as MMHg in the trench fauna were not accounted for in the 2 end member mixing model. If it was, the authors should make that more clear in the methods and code annotations. Such a distinction must be outlined clearly if the authors wish to support their conclusion that anthropogenic Hg is efficiently transported to depth since the low percent THg as MMHg in the measured samples is generally low and MMHg from the 2 end members could therefore be greatly overestimated.

Additional line comments:
P2 L45: DMHg is often measured at comparable concentrations with MMHg (Bowman et al., GEOTRACES sections; Munson et al., N. Pacific stations referenced in SI).

P3 L57: Awkward second sentence. “Yet, available data show…” would be better.

P3 L59-61: I disagree that there are any additional technical challenges associated with deep waters. Typically, we have focused higher resolution sampling of Hg species and incubation studies from waters because of high MMHg proportions as dissolved oxygen decreases in depth of net remineralization or due to links with pelagic food webs.

P5 L97: The significantly negative correlation referred to (Fig S2B) is listed as being calculated than a larger sample size than is possible given the data in Table S1. This must be addressed.

P5 L106: As noted in the major points above, both the median (14 %) and mean (20 %) values for THg as MMHg are quite low relative to pelagic fish. Thus, the emphasis on MMHg cycling rather than THg cycling represents an overinterpretation of the data, since the majority of THg in these amphipod samples in present as Hg(II).

L171-174: More detail is needed to understand how the sediment collection technique provides a sufficient comparison to published sediment samples from the NPO. Were surface samples collected? How did they insure the samples were not subject to mixing?


Reviewer #3 (Remarks to the Author):

Review of NCOMMS-20-02063

Methylmercury produced in upper oceans accumulates in deep Mariana Trench fauna

Recommendation: accept with minor corrections

Comments:
This manuscript describes results of total and methylmercury concentration and mercury stable isotope analyses of amphipods, one fish, and sediment samples from the Mariana and Yap trenches in the North Pacific Ocean. The authors compare their measurements to measured and calculated values of stable mercury isotopes in particles and fish from the upper ocean (above 1000m). Based on similarity between d202Hg and Δ199Hg of the trench fauna and fish from 400-600m depth, as well as the ratio of 199:202 and 199:201, the authors conclude that methylmercury in the trench fauna originates in the upper ocean, rather than at depth, and is carried to the deep ocean through sinking particles. A simple mixing model was used to estimate percentages of MMHg coming from surface and intermediate waters and discuss the implications of the results on MMHg and DMHg formation in deep waters.

In general, the manuscript is well written and clearly thought out, and is a valuable contribution to the growing field of mercury stable isotopes. The conclusions are reasonable and appropriate given the data presented, and are consistent with other interpretations in the literature. The figures are easily legible and support the conclusions. There is a lot of interesting data in the supplemental file, which is very helpful, but I wonder if some of it should be included in the main manuscript rather than the supplement. Mercury stable isotopes can be difficult to understand and interpret, but I think the authors in general did a nice job describing how they came to their conclusions. I have included specific comments following that I think will help to improve an already decent manuscript.

Another general comment would be to define and be consistent with your depth definitions. In the introduction you refer to the epipelagic, mesopelagic, and deep waters, but later in the manuscript you refer to upper, surface, and intermediate without indicating if they are the same or different depth ranges.

Specific comments:

Abstract: I would add in your estimates of surface and intermediate contributions to deep MMHg

Line 34: define MMHg here
36: releases; delete comma between water and eventually
55: delete “the” prior to field
57: delete “yet”
65: do you want “even-MIF”, rather than “even-MF”?
67: skeptically seems like an odd word choice. Do you mean “proposed to be”
70: potential; delete “and the”
Figure 1: explain what a, b, and c are in the figure caption, which one is Mariana versus Yap, etc. Add N and E designators to the lat lon axes.
86-87: replace “have a mean value” with “average”
88-89: suggest adding in the values from the other studies so the reader can make their own comparison without having to look up those papers.
89: Suggest saying “The relatively high concentrations”
90-91: I’m not sure if you mean that only half of the total number of samples had enough mass to even try measuring MMHg, or if only half of the ones that were measured had a concentration above the detection limit. Please clarify.
96: suggest replacing “and” with “or with” prior to body lengths
97-99: have there been other observation of in-vivo demethylation that you can reference?
104: range
106: other studies have seen relationships between %MMHg and isotope values. Any idea why you might not have seen it here? Is it just because the variation is small?
108: Tables S1 and S2 don’t show the relationships that you are referring to, just the raw data. Consider whether you want to add in graphs of the listed variables so the readers can see the lack of relationship themselves.
131: “messages” feels like an odd word choice…do you mean processes, or perhaps insight into Hg cycling?
160-176: This whole paragraph is clear and well-presented and the conclusions seem reasonable; nice work!
175: here is a use of “surface” that may not be what you actually mean. To me, surface is a very shallow layer (0-2m), but methylation in the upper layers is posited in some of your citations and in your intro etc. to occur below the photic zone, which is not what I think of when I think of surface water. If you can be more specific with your depths that would be helpful.
Figure 4: I really like what you are trying to do with this figure, but I find it a little hard to interpret still. Could you number the different processes and/or somehow indicate which one contributes to the odd-MIF proportion? For example, in the conversion from Hg(II) to MMHg on the top line, is it possible to indicate in the figure that the difference in odd-MIF between Hg(II) and MMHg is due to demethylation rather than methylation? It’s stated in the text, but I think it would be even better if it was also indicated in the figure itself.
213: delete “we notice that”
239-252: I think this discussion of DMHg formation is interesting, but it seems to come out of nowhere. Is it relevant to the trench fauna somehow? If so, mention it in the introduction. If it’s just informative regarding Hg cycling in the ocean, it could be tied in to the rest of the discussion a little better.
265: Explain why you separated tissues, and why you removed the heads, please.
335-342: Why did you use fish tissues for the surface but particles for the intermediate depths? If you used fish values for both zones (or particles), would the weighted means come out the same?
Methods in general: please indicate what program and what tests you used to evaluate the correlations and any other statistical tests you performed.

Supplementary Information:
Thanks for including photos of the biota!
Fig S4: X axis label should be “total”
Table S1: Does individual number refer to the number of individuals per sample? Could be clearer. I would also state somewhere here that the concentrations are on a dry weight basis.

**Author rebuttal, first version:**

Reviewer #1 (Remarks to the Author):

Methylated Hg in the deep ocean, especially DMHg, has been given very little attention by the oceanographic community, with no study reporting beyond concentration values. This study provides a novel investigation of the source of MMHg to deep-sea biota, while providing new insight on the source of DMHg to the deep ocean water column. The results of this study show that MMHg produced in the surface ocean from anthropogenic Hg affects not only epipelagic and mesopelagic organisms, but deep-sea ecosystems as well. This study is also of high value to the modeling community, as increased efforts and data are needed to improve models of methylated Hg in the global ocean. A few minor edits are suggested below.

We thank the reviewer for the encouraging comments.

Line 45: This statement needs to be supported by a reference. A lot of studies in the Pacific report equal amounts of MMHg and DMHg in the water column.

Four references including three for Pacific Ocean’s and one for Atlantic Ocean’s MMHg and DMMHg have been added in the updated manuscript.

Hammerschmidt, C. R. & Bowman, K. L. Vertical methylmercury distribution in the subtropical North Pacific Ocean. Mar. Chem. 132-133, 77-82 (2012).

Bowman, K. L., Hammerschmidt, C. R., Lamborg, C. H. & Swarr, G. Mercury in the North Atlantic Ocean: The U.S. GEOTRACES zonal and meridional sections. Deep-Sea Res. PT. II 116, 251-261 (2015).

Munson, K. M., Lamborg, C. H., Swarr, G. J. & Saito, M. A. Mercury species concentrations and fluxes in the Central Tropical Pacific Ocean. Global Biogeochem. Cycles 29, 656-676 (2015).

Bowman, K. L., Hammerschmidt, C. R., Lamborg, C. H., Swarr, G. J. & Agather, A. M. Distribution of mercury species across a zonal section of the eastern tropical South Pacific Ocean (U.S. GEOTRACES GP16). Mar. Chem. 186, 156-166 (2016).

Line 67: The authors should elaborate on “skeptically related” if this statement is to remain in

the manuscript.

“*skeptically related*” has been changed to “*proposed to be related*”.

Line 225: There is a new paper on Hg cycling genes in the ocean that supports the discussion here (Bowman et al. 2019; Limnology & Oceanography). The authors found no evidence of hgcAB in marine waters, but did find merB in the deep ocean.

We now include appropriate acknowledgement of this critical study in the revised manuscript as Ref #58.

R3

Reviewer #2 (Remarks to the Author):

The Sun et al. study uses stable isotope signatures of Hg in North Pacific deep trench amphipods and marine sediment in comparison to previously published data from surface and intermediate ocean depths to assess MMHg cycling and sources in deep water. The study identifies sinking particles formed in the upper ocean as the primary source of MMHg in the deep ocean, rather than in situ production, and uses Monte Carlo simulations to determine the overall sources MMHg in deep trench fauna from surface and intermediate sources.

1. The application of isotope signatures to deep sea organisms seems a natural progression based on the last decade or so of marine Hg literature. The emphasis on sinking particles as the Hg source to deep sea amphipods does not appear novel. Why the authors assume that sediments would be a potential food source for freely moving amphipods captured from bait traps is unclear to me. Evidence that these amphipods can feed from sediment would support the authors' claims that Hg from surface particles are more bioavailable than Hg in sediments. From the reviewer’s comments we conclude that we have not explained our arguments and deductions well enough. We see two points of confusion:

1. We concur with the reviewer’s comment that “Hg transport by sinking particles to deep sea amphipods is not a very novel finding”. In that statement, Hg refers to total Hg, which is predominantly inorganic Hg(II). Like other inorganic trace metals (Fe, Zn, Cu...), inorganic Hg(II) is transported to depth by sinking particles. However, in our study we focus on the rapid transport of upper ocean MMHg, the organometallic cation, by sinking particles, which we find to be the main MMHg source of deep biota. The MMHg cation is much less particle reactive than divalent inorganic Hg(II), and generally not considered (modeled) to be transported to depth by sinking particles. Current paradigms on marine MMHg cycling assume that a) MMHg is produced by anaerobic bacteria (e.g., sulfate reducing bacteria) in marine sediments, and diffuses to the overlying water column, and b) is produced in the deep water column itself. Our observation that deep fauna MMHg has a pure surface ocean isotope fingerprint refutes the current paradigm, and leaves only one option: MMHg is transported to depth, not as inorganic Hg(II), but in the form of MMHg to begin with, and most likely sorbed to sinking particles.
2. The reviewer asks “Why the authors assume that sediments would be a potential food source for freely moving amphipods is unclear to me”. What we mean to convey is not that sediments are a food source to amphipods, because they are not. We mean to say but that MMHg formed in sediments, and subsequently diffused to the overlying water column (a 50y old paradigm in Hg science), is not a MMHg source to amphipods. We stated that sinking particles are the potential food sources, as we stated in manuscript (3^rd^ paragraph of Discussion): “*The trench amphipods are benthic scavengers, which mainly reside in the top of the sediments surrounding the hydrothermal vent fields, and feed on sinking particles*”. So, the identification of food sources for amphipods is not our focus, although it is an interesting topic. We mainly focus on where MMHg in trench fauna comes from: upper ocean versus in-situ production. Our Hg

R4

isotope evidence shows that nearly all MMHg in trench fauna comes from the upper ocean, and this upper ocean MMHg likely transports to the deep ocean by sinking particles and then is accumulated in trench fauna. Regardless of (fresh) sediments or falling particles as the food sources, only the MMHg that traced back to upper ocean rather than that in-situ produced in sediments or falling particles is accumulated in trench fauna.

The primary conclusions of the study are based on analysis of THg and MMHg (typically % THg has MMHg) from collected fauna and marine sediments. My concerns over the conclusions drawn from the provided data include:

1. The statistical analysis presented in Fig S2B, Fig S4A, and Fig S4B are inconsistent with the data provided in Table S1 or Table S2. The length relationship displayed in Fig S2B cannot be based on 25 individual measurements, since only 11 amphipod samples were analyzed for MMHg. Likewise, the sample size given in Fig S4 is only possible if both the TY-22-3 duplicate and gut sample and the YW-12-4 lipid sample are counted as individual samples, which is an inaccurate description, as they represent either a measure of error (duplicate) or subsets of the whole body samples (gut/lipid). The authors need to verify their correlation calculations in order to correct their figures and the resulting interpretation in L95-100 and L114-L119.

We thank the reviewer for pointing out this inconsistency. We have carefully checked our data, updated these figures and their legends, and added more information for relevant interpretations (see Results). For Fig. S2B, the original regression calculation was correct, but there was a typo of sample number which should be 11 rather than 25. We have corrected this typo. For Fig S4 (now updated as Fig. 3 in main text), gut and lipid samples, but not duplicate TY-22-3 (which was already averaged with TY-22-3 as one sample), were included in the original regression calculation. We now remove the gut and lipid points, and recalculated the regression parameters in the updated figures. The recalculated regression parameters all support our previous conclusions and relevant interpretations.

2. The overall percent THg as MMHg in the measured samples (2-59 %) is low compared to fish. The mean THg as MMHg value is ~20 %, whereas the comparison to published fish data, in which MMHg often exceeds 90 % of the measured THg. Despite the fact that the majority of Hg in all but 1 sample (YW-9) is present as THg, the authors interpret the stable isotopic signatures as representing sources of MMHg, rather than THg. Thus, the focus on MMHg is poorly supported by the data, specifically in the following sections:
a. L134-135: The source allocation for deep ocean MMHg sources rather than THg.
b. L172-176: The discussion of trench MMHg rather than trench THg.
c. L189-191: The supposition that MMHg is transported to depth, rather than THg.
d. L193-195: The assessment of MMHg, rather than THg, sinking rates.
e. L217-225: Why MMHg degredation is invoked when the majority of the Hg isotopes represent those of THg.
f. L239-252: Any discussion of DMHg production at depth is dependent on the

R5

We understand the reviewer’s comment very well, and we regret we were not able to make clearer why the THg isotope compositions reflect exactly the MMHg isotope compositions. Our data shows evidence that inorganic Hg(II) (abbreviated as IHg(II)) in trench fauna is nothing other than the product of in-vivo demethylation of ingested MMHg, rather than bioaccumulated ambient IHg(II) from falling particles, sediments or deep seawater. This in-vivo MMHg demethylation causes very limited isotope fractionation between MMHg and IHg(II) (Kwon et al., 2012; Kwon et al., 2013). The total Hg isotope signatures measured in fauna are therefore those of accumulated MMHg, and it is thus reasonable that we interpret THg as MMHg. This is supported by the following evidence.

1) A significantly negative correlation is observed between MMHg% and body lengths (a surrogate of life spans) of amphipods (Fig. S2B, copied here below), suggesting that in-vivo demethylation of MMHg likely occurs in the amphipods, most likely in the intestinal tract. This trend is very well known from marine mammals (see Masbou et al., 2018).

2) The trench fauna MIF values exhibit a Δ^199^Hg/Δ^201^Hg regression slope that is strongly diagnostic of MMHg photodegradation rather than IHg(II) photoreduction (note only photochemical processes can produce MIF). Δ^199^Hg/Δ^201^Hg slope of the trench fauna (1.15 ± 0.08) is in excellent agreement with the slope (~1.2) reported for fishes (>90% of MMHg%) from coastal/open marine waters. IHg(II) photoreduction generally leads to Δ^199^Hg/Δ^201^Hg slopes of 1.00 ± 0.05. The observed slope of 1.15 on trench fauna therefore suggests that it is inherited from MMHg, following in-vivo demethylation.
3) Our data shows that all trench amphipods have very consistent, high δ^202^Hg (0.27 ± 0.14‰) and Δ^199^Hg (1.47 ± 0.13‰), and exhibit very small variation in both signatures in spite of large variation of MMHg% (Fig. 3). The snailfish with 83% of MMHg% has nearly the same Hg isotope compositions as trench amphipods, which have varying and low MMHg%. If the IHg(II) was to be assimilated from the surrounding environments, we would expect more scattered Hg isotope distribution due to the mixing of IHg(II) and MMHg. We would expect the trench fauna with high IHg(II)% to have low δ^202^Hg and Δ^199^Hg , because IHg(II) in the deep ocean (also the whole ocean) environment has relatively low δ^202^Hg and Δ^199^Hg , as seen from our measured seafloor sediments (δ^202^Hg = -0.96 ± 0.27‰, Δ^199^Hg = 0.20 ± 0.07‰) and previously reported

R6

open ocean particles (Motta et al., 2019: δ^202^Hg = -0.12 ± 0.13%, Δ^199^Hg = 0.16 ± 0.09%) and sediments (e.g., Gehrke et al., 2009: δ^202^Hg = -0.82 ± 0.17%, Δ^199^Hg = 0.08 ± 0.04%; Ogrinc et al., 2019: δ^202^Hg = -2.30 to 0.78%, Δ^199^Hg = 0.10 ± 0.04%) and seawater (δ^202^Hg = -1.73 ± 0.72%, Δ^199^Hg = 0.16 ± 0.10%) (Štrok et al., 2019; Štrok et al., 2014) that are primarily composed of IHg(II).

4) Little differences (<0.1%) in Hg isotope compositions are observed between the whole tissues and the separated tissues (muscle, lipid and gut contents) of amphipods, although their Hg concentrations differed significantly (Table S2). It means that the internal distribution or in-vivo transformation of Hg species do not significantly fractionate Hg isotopes between MMHg and IHg(II) in amphipods.

Gehrke, G.E., Blum, J.D. and Meyers, P.A. (2009) The geochemical behavior and isotopic composition of Hg in a mid-Pleistocene western Mediterranean sapropel. Geochim. Cosmochim. Acta 73, 1651-1665.

Kwon, S.Y., Blum, J.D., Carvan, M.J., Basu, N., Head, J.A., Madenjian, C.P. and David, S.R. (2012) Absence of Fractionation of Mercury Isotopes during Trophic Transfer of Methylmercury to Freshwater Fish in Captivity. Environ. Sci. Technol. 46, 7527-7534.

Kwon, S.Y., Blum, J.D., Chirby, M.A. and Chesney, E.J. (2013) Application of mercury isotopes for tracing trophic transfer and internal distribution of mercury in marine fish feeding experiments. Environ. Toxicol. Chem. 32, 2322-2330.

Masbou, J., Sonke, J.E., Amouroux, D., Guillou, G., Becker, P.R. and Point, D. (2018) Hg-Stable Isotope Variations in Marine Top Predators of the Western Arctic Ocean. ACS Earth Space Chem. 2, 479-490.

Motta, L.C., Blum, J.D., Johnson, M.W., Umhau, B.P., Popp, B.N., Washburn, S.J., Drazen, J.C., Benitez-Nelson, C.R., Hannides, C.C.S., Close, H.G. and Lamborg, C.H. (2019) Mercury cycling in the North Pacific Subtropical Gyre as revealed by mercury stable isotope ratios. Global Biogeochem. Cycles 33, 777-794.

Ogrinc, N., Hintelmann, H., Kotnik, J., Horvat, M. and Pirrone, N. (2019) Sources of mercury in deep-sea sediments of the Mediterranean Sea as revealed by mercury stable isotopes. Sci. Rep. 9, 11626.

Štrok, M., Baya, P.A., Dietrich, D., Dimock, B. and Hintelmann, H. (2019) Mercury speciation and mercury stable isotope composition in sediments from the Canadian Arctic Archipelago. Sci. Total Environ. 671, 655-665.

Štrok, M., Hintelmann, H. and Dimock, B. (2014) Development of pre-concentration procedure for the determination of Hg isotope ratios in seawater samples. Anal. Chim. Acta 851, 57-63.

Part of the above arguments have been already included in the original manuscript. To address the reviewer’s concern, we put all these arguments together in a new paragraph (2^nd^ paragraph in the Discussion before discussing fauna MMHg sources) of revised manuscript as follows.

*The isotope compositions of total Hg in trench amphipods are representative of the isotope*

R7

*signatures of MMHg in the same amphipods, despite the relatively low MMHg fractions of 2-59%. The reason for this is that the IHg(II) in the amphipods is likely produced by in-vivo degradation of MMHg rather than assimilation of IHg(II) from the ambient environments. This is supported by multiple lines of evidence. 1) The significantly negative correlation between MMHg% and body length of amphipods (Fig. S2b) indicates that in-vivo demethylation of MMHg likely occurs in the amphipods. This trend is well known from marine mammals (Masbou et al., 2018). 2) The trench fauna MIF values exhibit a Δ^199^Hg/Δ^201^Hg regression slope of 1.15 that is diagnostic of MMHg photodegradation rather than IHg(II) photoreduction. This suggests that the IHg(II) fraction in amphipods inherits its Δ^199^Hg and Δ^201^Hg signatures from ingested MMHg, following in-vivo demethylation. 3) Trench amphipods have very consistent, high δ^202^Hg (0.27 ± 0.14‰, 1SD) and Δ^199^Hg (1.47 ± 0.13‰, 1SD), and exhibit little variation in both signatures in spite of large variations in MMHg% (Fig. 3). The snailfish with 83% of MMHg% has nearly the same Hg isotope composition as trench amphipods which have varying and low MMHg%. If IHg(II) was to be assimilated from the surrounding environments, we would expect a more scattered Hg isotope distribution due to the mixing of IHg(II) and MMHg. We would expect that the trench fauna with high IHg(II)% to have low δ^202^Hg and Δ^199^Hg, because IHg(II) in the oceans has relatively low δ^202^Hg and Δ^199^Hg, as seen from our measured seafloor sediments and previously reported open ocean particles (Motta et al., 2019) and sediments (Gehrke et al., 2009; Ogrinc et al., 2019), and coastal seawater (Štrok et al., 2015; Štrok et al., 2014). 4) Insignificant differences (<0.1‰) in Hg isotope compositions are observed between the whole tissues and the separated tissues (muscle, lipid and gut contents) of amphipods, although their Hg concentrations and speciation vary significantly (Table S2). This suggests that the internal distribution or in-vivo transformation of Hg species do not significantly fractionate Hg isotopes between MMHg and IHg(II) in amphipods. In the following we discuss the origin of fauna MMHg, based on all available open ocean Hg isotope data, and make a number of deductions that constrain the deep ocean MMHg cycling.*

3. The sediment samples are bulk samples from box cores, with no information provided concerning the sediment horizon captured (these mixed sediments may be far from newly deposited sediment from falling particles). The authors compare these directly to the collection of suspended sediment samples collected from in situ pumps in intermediate waters, which by definition collect primarily neutrally buoyant, rather than sinking particles. Furthermore, only THg analysis was performed on the sediment samples and if the percent THg as MMHg in these sediments is lower than zooplankton, often < 1 % in marine sediments, the isotopic signatures of the sediment poorly represent those of MMHg.

Ocean floor sediments were collected with a box corer that was launched by a geological winch. What we analyzed are the top 0-6 cm layer of box sediments (approximate migration range of amphipods, added in the Sampling section: *Immediately after recovery, short sediment cores were collected by pushing PVC tubes (L = 80 cm, ID = 9 cm) into the sediment blocks. The top 0-6 cm layers of sediments from all the short cores were then subsampled and stored at -20 °C before freeze-drying and homogenization*). As we argued in

R8

the general comment #1, the identification of food sources is not our focus. We mainly focus on where MMHg in trench fauna comes from: upper ocean versus *in-situ* production.

The reviewer questions whether the isotope signatures of THg in sediments, i.e. 99% IHg(II), represent the isotope signatures of MMHg formed from IHg(II) in sediments. That is an understandable question, and the answer is ‘mostly no’ for δ^202^Hg, but it is ‘yes’ for MIF signatures (Δ^199^Hg, Δ^200^Hg, and Δ^201^Hg). The reason is that MIF signatures can only dramatically change by photochemistry. As there is no light in the trench, any MMHg formed in-situ, inherits the MIF signature of IHg(II) substrate. In other words, if abundant Hg methylation in deep trench sediments was to supply the water column and fauna with MMHg, then that MMHg should have inherited the low MIF signatures of local IHg(II) in sediments. This is clearly not the case: deep fauna has MIF signatures straight imported from photodegraded MMHg in the photic zone, which is a real surprise.

A thorough critique of the Monte Carlo simulations falls outside my field of expertise. However, from reviewing the code, it appeared to me that the percentage THg as MMHg in the trench fauna were not accounted for in the 2 end member mixing model. If it was, the authors should make that more clear in the methods and code annotations. Such a distinction must be outlined clearly if the authors wish to support their conclusion that anthropogenic Hg is efficiently transported to depth since the low percent THg as MMHg in the measured samples is generally low and MMHg from the 2 end members could therefore be greatly overestimated.

Please refer to our response to your general comment #2. In summary, we present strong evidence to show that the total Hg isotope signatures measured in fauna are in fact those of accumulated MMHg, and it is justified that we can treat THg as MMHg. We now add a comment to the mixing model text to clarify this “*Since total Hg in the trench fauna is derived from MMHg in the upper ocean...*”.

Additional line comments:

P2 L45: DMHg is often measured at comparable concentrations with MMHg (Bowman et al., GEOTRACES sections; Munson et al., N. Pacific stations referenced in SI).

We now change “major fraction” to “large fraction”, and add these references in the revised manuscript.

P3 L57: Awkward second sentence. “Yet, available data show...” would be better. We now change it to “Yet, available data show...”.

P3 L59-61: I disagree that there are any additional technical challenges associated with deep waters. Typically, we have focused higher resolution sampling of Hg species and incubation studies from waters because of high MMHg proportions as dissolved oxygen decreases in depth of net remineralization or due to links with pelagic food webs.

We concur with the reviewer’s point, and now change this sentence as “*Due to sparse incubation and measurement of deep waters...*”.

R9

P5 L97: The significantly negative correlation referred to (Fig S2B) is listed as being calculated than a larger sample size than is possible given the data in Table S1. This must be addressed. Please refer to our response to your general comment #1.

P5 L106: As noted in the major points above, both the median (14 %) and mean (20 %) values for THg as MMHg are quite low relative to pelagic fish. Thus, the emphasis on MMHg cycling rather than THg cycling represents an overinterpretation of the data, since the majority of THg in these amphipod samples in present as Hg(II).

Please refer to our response to your general comment #2.

L171-174: More detail is needed to understand how the sediment collection technique provides a sufficient comparison to published sediment samples from the NPO. Were surface samples collected? How did they insure the samples were not subject to mixing?

Please refer to response to your general comment #3. Since we analyzed the top 0-6 cm layer of box sediments, slight mixture caused by bioturbation if existed is not critical issue. As we argued before, the suspended and deposited particle IHg(II) across the ocean appears to have consistent Hg isotope values, especially MIF. The reason we sampled and measured sediments is not to anchor the food sources but to infer the Hg MIF signatures of in-situ produced MMHg.

Reviewer #3 (Remarks to the Author):

Review of NCOMMS-20-02063

Methylmercury produced in upper oceans accumulates in deep Mariana Trench fauna Recommendation: accept with minor corrections

Comments:

This manuscript describes results of total and methylmercury concentration and mercury stable isotope analyses of amphipods, one fish, and sediment samples from the Mariana and Yap trenches in the North Pacific Ocean. The authors compare their measurements to measured and calculated values of stable mercury isotopes in particles and fish from the upper ocean (above 1000m). Based on similarity between d202Hg and Δ199Hg of the trench fauna and fish from 400-600m depth, as well as the ratio of 199:202 and 199:201, the authors conclude that methylmercury in the trench fauna originates in the upper ocean, rather than at depth, and is carried to the deep ocean through sinking particles. A simple mixing model was used to estimate percentages of MMHg coming from surface and intermediate waters and discuss the implications of the results on MMHg and DMHg formation in deep waters.

In general, the manuscript is well written and clearly thought out, and is a valuable contribution to the growing field of mercury stable isotopes. The conclusions are reasonable and appropriate given the data presented, and are consistent with other interpretations in the literature. The figures are easily legible and support the conclusions. There is a lot of interesting data in the supplemental file, which is very helpful, but I wonder if some of it should be included in the

R10

main manuscript rather than the supplement. Mercury stable isotopes can be difficult to understand and interpret, but I think the authors in general did a nice job describing how they came to their conclusions. I have included specific comments following that I think will help to improve an already decent manuscript.

We thank the reviewer for the encouraging comments. We have moved the original Fig. S4 in SI (relationships between Hg isotope values and MMHg%) to the main text as the new Fig. 3.

Another general comment would be to define and be consistent with your depth definitions. In the introduction you refer to the epipelagic, mesopelagic, and deep waters, but later in the manuscript you refer to upper, surface, and intermediate without indicating if they are the same or different depth ranges.

We have made a consistent definition for different types of ocean waters and layers (i.e., epipelagic waters from surface ocean at 0-100 m, mesopelagic waters from intermediate ocean at 100-1000 m, and deep waters from deep ocean below 1000 m; the upper ocean includes surface and intermediate ocean) at their first appearance.

1) In the Abstract “*...Recent studies show abundant methylated Hg in deep oceans (>1000 m), yet its origin remains uncertain... Given the identical odd-MIF in trench fauna and North Pacific upper ocean (<1000 m) biota MMHg, ...We estimate that 37-48% and 52-63% of MMHg in the Mariana fauna is respectively sourced from surface (0-100 m) and intermediate (100-1000 m) oceans...*”.
2) In the end of 1^st^ paragraph of Introduction “*This so-called biological pump results in a macronutrient-like vertical distribution of total Hg concentrations, with low values in epipelagic waters of the surface ocean (0-100 m), increasing through oxygen-depleted mesopelagic waters of the intermediate ocean (100-1000 m), and remaining high in deep ocean waters (>1000 m)*”.
3) *Fig. 2 also shows the depth ranges of surface, intermediate and deep oceans.*

Specific comments:

Abstract: I would add in your estimates of surface and intermediate contributions to deep MMHg

The sentence *“We estimate that 37-48% and 52-63% of MMHg in the Mariana fauna is respectively sourced from surface (0-100 m) and intermediate (100-1000 m) oceans”* is now added in the abstract.

Line 34: define MMHg here

The full name of MMHg (monomethylmercury) is now defined here.

36: releases; delete comma between water and eventually
Corrected.

55: delete “the” prior to field

R11

Deleted.

57: delete “yet”

We now change it to “*Yet, available data show...*”.

65: do you want “even-MIF”, rather than “even-MF”? Yes. This typo has been corrected.

67: skeptically seems like an odd word choice. Do you mean “proposed to be” Yes. We now change “*skeptically*” to “*proposed to be*”.

70: potential; delete “and the”
Corrected.

Figure 1: explain what a, b, and c are in the figure caption, which one is Mariana versus Yap, etc. Add N and E designators to the lat lon axes.

The Fig. 1 and its caption are now updated to reflect a, b and c and the directions of latitude-longitude.

86-87: replace “have a mean value” with “average”
Replaced.

88-89: suggest adding in the values from the other studies so the reader can make their own comparison without having to look up those papers.

We have added the values cited from other studies, now this sentence reads as “*Total Hg concentrations in trench amphipods have an average of 547 ± 230 ng g^-1^ (1SD, 235 to 1070 ng g-1, dry weight, Table S1), which is comparable to their counterparts from the abyssal Arctic Ocean (250 to 1000 ng g^-1^), but considerably higher than benthic organisms in freshwater and coastal ecosystems (mostly <400 ng g^-1^ on average)*”.

89: Suggest saying “The relatively high concentrations”
Changed.

90-91: I’m not sure if you mean that only half of the total number of samples had enough mass to even try measuring MMHg, or if only half of the ones that were measured had a concentration above the detection limit. Please clarify.

We only measured about half of samples for MMHg concentrations, because 1) this amount of data are sufficient to support our interpretations and 2) the remaining half of samples were almost exhausted after Hg isotope analysis. We now thus change this sentence as “*MMHg concentrations were measured in nearly half of amphipods, ...*” to avoid confusion.

96: suggest replacing “and” with “or with” prior to body lengths

R12

We have changed this sentence as “*Total Hg concentrations of amphipods show no correlation with their depths of occurrence (R^2^ = 0.00, P = 0.85), and an insignificantly positive correlation with their body length, a surrogate of life span (R^2^ = 0.12, P = 0.08, Fig. S2a)*”.

97-99: have there been other observation of in-vivo demethylation that you can reference? This in-vivo demethylation is well known from marine mammals (Masbou et al., 2018) (added in the 2^nd^ paragraph of Discussion). Here, We think the in-vivo demethylation may relate to the gut microbiota of amphipods, since we show that microbial degradation of MMHg likely occur in deep oceans (5^th^ paragraph of Discussion) and a recent a recent study (Bowman et al. 2020) found merB in deep oceans. In the 5^th^ paragraph of Discussion, we now add a sentence “*The bacteria in deep oceans that are capable to demethylate MMHg also likely exist in the guts of trench amphipods, and cause the in-vivo demethylation of MMHg we infer above*” to explain the proposed in-vivo demethylation.

Bowman K. L., Collins R. E., Agather A. M., Lamborg C. H., Hammerschmidt C. R., Kaul D., Dupont C. L., Christensen G. A. and Elias D. A. Distribution of mercury-cycling genes in the Arctic and equatorial Pacific Oceans and their relationship to mercury speciation [J]. Limnol. Oceanogr. 2020; 65: S310-S320.

Masbou, J., Sonke, J.E., Amouroux, D., Guillou, G., Becker, P.R. and Point, D. (2018) Hg-Stable Isotope Variations in Marine Top Predators of the Western Arctic Ocean. ACS Earth Space Chem. 2, 479-490.

104: range
Corrected.

106: other studies have seen relationships between %MMHg and isotope values. Any idea why you might not have seen it here? Is it just because the variation is small?

The reason for %MMHg vs. isotope values relationships seen in other studies is because their studied biota assimilate inorganic Hg(II) and MMHg from ambient environments which have quite different isotope values. The end-member mixture of varying proportions of inorganic Hg(II) and MMHg results in these relationships.

However, in our study, these relationships are absent. This is because the biotic Hg(II) is derived from in-vivo demethylation of ingested MMHg by trench fauna rather than inorganic Hg(II) from ambient environments (falling particles or sediments or seawater). This in-vivo MMHg demethylation causes very limited isotope fractionation between MMHg and Hg(II). Thus, we observe very limited isotope variation in trench fauna in spite of large variation of MMHg%. We present four lines of evidence to support this viewpoint, in our response to reviewer 2’s general comment #2 above. In the revised manuscript we have clarified the text to explain this better (see 2^nd^ paragraph of Discussion).

R13

108: Tables S1 and S2 don’t show the relationships that you are referring to, just the raw data. Consider whether you want to add in graphs of the listed variables so the readers can see the lack of relationship themselves.

We now add a new figure S3 in SI to show the correlations of Hg isotope compositions with total Hg concentrations, body lengths, depths of occurrence and sampling dates of Mariana/Yap trench amphipods, and modify the text accordingly (2^nd^ paragraph of Results).

131: “messages” feels like an odd word choice...do you mean processes, or perhaps insight

into Hg cycling?

We now replace “*messages*” with “*insight into hadal Hg provenance*”

160-176: This whole paragraph is clear and well-presented and the conclusions seem

reasonable; nice work!

We extend our thanks to the reviewer’s comments.

175: here is a use of “surface” that may not be what you actually mean. To me, surface is a very shallow layer (0-2m), but methylation in the upper layers is posited in some of your citations and in your intro etc. to occur below the photic zone, which is not what I think of when I think of surface water. If you can be more specific with your depths that would be helpful.

Please refer to our response to your general comment.

Figure 4: I really like what you are trying to do with this figure, but I find it a little hard to interpret still. Could you number the different processes and/or somehow indicate which one contributes to the odd-MIF proportion? For example, in the conversion from Hg(II) to MMHg on the top line, is it possible to indicate in the figure that the difference in odd-MIF between Hg(II) and MMHg is due to demethylation rather than methylation? It’s stated in the text, but I think it would be even better if it was also indicated in the figure itself.

Good suggestion! Different processes have been numbered in the updated Figure 5, and explained in the caption. We also add MIF labels for Hg(II) photoreduction and MMHg photodegradation in this figure.

213: delete “we notice that”
Deleted.

239-252: I think this discussion of DMHg formation is interesting, but it seems to come out of nowhere. Is it relevant to the trench fauna somehow? If so, mention it in the introduction. If it’s just informative regarding Hg cycling in the ocean, it could be tied in to the rest of the discussion a little better.

Although the discussion on DMHg formation does not directly relate to our study, it elaborates on the implication of our study because we find that deep ocean MMHg is supplied from the upper ocean by particle sinking rather than in-situ microbial methylation (already added into the text of this section). Here, we tie this section with previous discussion by saying “*Our*

R14

*findings not only inform on the source of MMHg in the deep ocean, but also provide insight for the production of DMHg in the deep ocean*”. We feel that this DMHg section, although informative and speculative, could inspire future study of DMHg cycling in the oceans. Reviewer 1 made an explicit positive statement regarding our deduction on DMHg. We have taken up the suggestion to mention DMHg more in detail in the 2^nd^ paragraph of Introduction.

265: Explain why you separated tissues, and why you removed the heads, please.

The heads were removed to avoid potential contamination by bait, although this contamination is unlikely since we enclosed the bait tightly, and the captured amphipods mostly had empty gut contents. Tissues were separated for three large-size amphipod samples to assess whether internal distribution or in-vivo transformation of Hg species induce Hg isotope fractionation. The above information has been added in the Sampling section of revised manuscript.

335-342: Why did you use fish tissues for the surface but particles for the intermediate depths? If you used fish values for both zones (or particles), would the weighted means come out the same?

No, the weighted means would be much higher if we use fish tissues, and much lower if we use particles for both zones, compared with currently estimated values. We think it is not theoretically correct to use fish tissues or particles for both zones to calculate the weighted means of upper ocean MMHg isotope compositions. The fish tissues from the surface ocean integrate the photodegraded MMHg isotope signatures in surface seawater, while the fish tissues from the intermediate ocean integrate the mixed isotope signatures between photodegraded surface MMHg and non-photodegraded intermediate MMHg, due to the downward MMHg transport from surface to intermediate oceans (see our Fig. 5 or Blum et al. 2013). The original isotope signatures of non-photodegraded in-situ MMHg in the intermediate seawater without mixture are theoretically imparted from those of particle Hg(II), because MMHg is thought to be microbially produced from particle-supplied Hg(II). This is why we use fish tissues from the surface ocean and particles from the intermediate ocean to calculate the weighted means of upper ocean MMHg isotope compositions.

Blum, J.D., Popp, B.N., Drazen, J.C., Anela Choy, C. and Johnson, M.W. (2013) Methylmercury production below the mixed layer in the North Pacific Ocean. Nat. Geosci. 6, 879-884.

Methods in general: please indicate what program and what tests you used to evaluate the correlations and any other statistical tests you performed.

We now add a separate subsection “Description of statistical analysis” In the Methods to describe the statistical analysis used in the manuscript.

Supplementary Information:

Thanks for including photos of the biota!

We are very glad you like them.

R15

Fig S4: X axis label should be “total”
Corrected.

Table S1: Does individual number refer to the number of individuals per sample? Could be clearer. I would also state somewhere here that the concentrations are on a dry weight basis. Yes, we have changed “individual number” to “number of individuals per sample”. In Tables S1 and S2, “dry weight” was annotated in the total Hg and MMHg concentration columns.

END

R16

**Reviewer comments, second version:**

Reviewer #2 (Remarks to the Author):

Sun et al. have greatly improved the quality of the manuscript in their resubmission and have addressed both the statistical errors in their initial submission and improved their interpretation and presentation of the isotopic data.


Minor line comments (numbers refer to revision, not the version with tracked changes):
L 29: comma is not needed.
L 92: do not invoke significance with a sample size of one snailfish.
L 155: what is the mechanistic connection between demethylation in marine mammals and that in amphipods? If the authors wish to rely on the Masbou et al., 2018 reference, they must provide a link or, otherwise, cite a more appropriate reference.
L 189-191: a residence time of "thousands of years" is not unusual for seawater chemical species. Provide reference for the claim that this is "unlikely" or more closely link to the following sentence if you rely on patterns from NPO fish studies.
L 231-232: provide a reference for gut methylation.
L 245-246: I suggest: "in the deep ocean, but allow us to speculate about the production of DMHg" based on the lack of direct DMHg measurements, and the possibility that MMHg is a degradation product from DMHg (currently not in vogue, but Mason and Fitzgerald, 1993, etc.)


Reviewer #3 (Remarks to the Author):

Thank you for your thoughtful responses. I appreciate the clarification and found the majority of your edits in response to my comments to be sufficient. I have two queries below, the first of which, if I am interpreting everything correctly, would not require and additional modifications. The second is relevant to Reviewer 2’s critiques, which I think could be addressed further.

Original comment and response:
335-342: Why did you use fish tissues for the surface but particles for the intermediate depths? If you used fish values for both zones (or particles), would the weighted means come out the same?
No, the weighted means would be much higher if we use fish tissues, and much lower if we use particles for both zones, compared with currently estimated values. We think itis not theoretically correct to use fish tissues or particles for both zones to calculate the weighted means of upper ocean MMHg isotope compositions. The fish tissues from the surface ocean integrate the photodegraded MMHg isotope signatures in surface seawater, while the fish tissues from the intermediate ocean integrate the mixed isotope signatures between photodegraded surface MMHg and non-photodegraded intermediate MMHg, due to the downward MMHg transport from surface to intermediate oceans(see our Fig. 5 or Blum et al. 2013). The original isotope signatures of non-photodegraded in-situ MMHg in the intermediate seawater without mixture are theoretically imparted from those of particle Hg(II), because MMHg is thought to be microbially produced from particle-supplied Hg(II). This is why we use fish tissues from the surface ocean and particles from the intermediate ocean to calculate the weighted means of upper ocean MMHg isotope compositions.

Current comment:
The reasoning for using fish from the surface ocean makes sense to me, as you are only interested in MMHg and they are mostly MMHg. I just want to make sure that I am interpreting the use of particles for the intermediate ocean correctly. In theory, particles in the intermediate ocean would be bound to both IHg(II) and MMHg…some of the MMHg would have been produced in the upper ocean and have a photodegraded MMHg signature (relatively high MIF signature) and the rest would have a low MIF signature due to microbially mediated production at intermediate depths from IHg(II). In order to estimate this microbially produced MMHg MIF signature, you are using intermediate depth particles. Is the assumption that because %MMHg of Hg bound to particles is quite low, that the amount of photodegraded MMHg bound to sinking particles captured in the intermediate waters is not high enough to bias this estimate (what you are essentially measuring is the IHg(II) signature and using that as a proxy for intermediate water MMHg signature after correcting for the methylation shift)? If I am interpreting that correctly, using fish for surface and particles for intermediate makes sense.

However, that is then relevant to Reviewer 2’s major critique, and my previous question as shown below:

Original comment and response:
106: other studies have seen relationships between %MMHg and isotope values. Any idea why you might not have seen it here? Is it just because the variation is small?
The reason for %MMHg vs. isotope values relationships seen in other studies is because their studied biota assimilate inorganic Hg(II) and MMHg from ambient environments which have quite different isotope values. The end-member mixture of varying proportions of inorganic Hg(II) and MMHg results in these relationships.
However, in our study, these relationships are absent. This is because the biotic Hg(II) is derived from in-vivo demethylation of ingested MMHg by trench fauna rather than inorganic Hg(II) from ambient environments(falling particles or sediments or seawater). This in-vivo MMHg demethylation causes very limited isotope fractionation between MMHg and Hg(II). Thus, we observe very limited isotope variation in trench fauna in spite of large variation of MMHg%. We present four lines of evidence to support this view point, in our response to reviewer 2’s general comment #2 above. In the revised manuscript we have clarified the text to explain this better (see 2ndparagraph of Discussion).

Current comment:
Assuming the amphipods are consuming particles transporting both MMHg and IHg(II), but mostly IHg(II) as assumed above, it seems difficult to then assume that IHg(II) in the tissues is there solely from demethylation of ingested MMHg and they are not assimilating any IHg(II) from their food. The end member of ingested IHg(II) should somewhat similar to the intermediate water particle signature, if I am thinking this through correctly, and you assume no fractionation during trophic transfer. I agree with your arguments that demethylation is likely occurring, and that the MMHg is of surface origin, which would result in IHg(II) of similar signature for 199, with a slight shift of remaining MMHg for 202 if microbially demethylated, but…is there any way that particle bound IHg(II) transported to the deep water and ingested by the amphipods could have a similar signature to the MMHg…some combination of photochemical transformation and then ligand binding in the upper ocean (this would probably affect 202 most)? Or, mixing of particle signature and demethylated signature in vivo? Alternatively, is there any evidence you could present that excretion of ingested IHg(II) occurs at a greater rate than excretion of IHg(II) produced in vivo, which would result in retention and thus measurement of just the IHg(II) produced by demethylation (thus resulting in similar THg signatures across the range of %MMHg)? Is there evidence that bioavailable IHg(II) isotopic signature is closer to that of MMHg than to environmental IHg(II)? While you did add a reference regarding in vivo demethylation in marine mammals, I think it is important to demonstrate whether this has also been observed in crustaceans in other environments or not, if you are arguing that it is a critical process in driving observed isotope signatures. I don’t disagree with your interpretation, particularly as it relates to MMHg production in the upper ocean and transport to the hadal food web, but I wonder if there are other ways to explain why the IHg(II) and MMHg within the amphipod tissues have the same general signature (resulting in no relationship with %MMHg) that you could rule out and strengthen your arguments?

**Author rebuttal, second version:**

Reviewer #2 (Remarks to the Author):
Sun et al. have greatly improved the quality of the manuscript in their resubmission and have addressed both the statistical errors in their initial submission and improved their interpretation and presentation of the isotopic data.
Minor line comments (numbers refer to revision, not the version with tracked changes):
L 29: comma is not needed.

The comma is now deleted.

L 92: do not invoke significance with a sample size of one snailfish.

‘significantly higher’ is now changed to ‘higher’.

L 155: what is the mechanistic connection between demethylation in marine mammals and that in amphipods? If the authors wish to rely on the Masbou et al., 2018 reference, they must provide a link or, otherwise, cite a more appropriate reference.

Masbou et al., 2018 is cited here to show that the observed trend in amphipods has also been seen in mammals where this trend is interpreted as in-vivo MMHg demethylation. We now add a reference (Nolde et al., 2005) to observed MMHg demethylation in isopods, a close relative of amphipods. Similar studies have observed demethylation in fish and other animal guts (see review of Li et al., 2019). Basically, any organism with a gastro-intestinal tract can be expected to host MMHg demethylating microbes.

We now change this sentence as “*MMHg demethylation is commonly observed in organisms with a gastro-intestinal tract including isopods, fish and mammals*”, and cite Nolde et al. (2005), Li et al. (2019) in addition to Masbou et al. (2018).

Li, H., Lin, X., Zhao, J., Cui, L., Wang, L., Gao, Y., Li, B., Chen, C. and Li, Y.-F. (2019) Intestinal Methylation and Demethylation of Mercury. Bull Environ Contam Toxicol 102, 597-604.

Nolde, N., Drobne, D., Horvat, M. and Jereb, V. (2005) Reduction and methylation of mercury in the terrestrial isopod Porcellio scaber (Crustacea) and its environment. Environ. Toxicol. Chem. 24, 1697-1704.

L 189-191: a residence time of "thousands of years" is not unusual for seawater chemical species. Provide reference for the claim that this is "unlikely" or more closely link to the following sentence if you rely on patterns from NPO fish studies.

According to a review paper of Fitzgerald et al. (2007), it concluded that “It is also evident that the aqueous lifetimes for both MMHg and DMHg are short compared to ocean mixing time scales (500-1000 years)”. We have added this reference in the revised manuscript.

Fitzgerald, W.F., Lamborg, C.H. and Hammerschmidt, C.R. (2007) Marine biogeochemical cycling of mercury. Chem. Rev. 107, 641-662.

L 231-232: provide a reference for gut methylation.

We now cite a review paper of Li et al. (2019) on gut methylation and demethylation,and a paper of Nolde et al. (2005) for MMHg demethylation in isopods.

L 245-246: I suggest: "in the deep ocean, but allow us to speculate about the production of DMHg" based on the lack of direct DMHg measurements, and the possibility that MMHg is a degradation product from DMHg (currently not in vogue, but Mason and Fitzgerald, 1993, etc.)
We thank the review’s suggestion, and now change this sentence as “*Our findings not only inform on the source of MMHg in the deep ocean, but also allow us to speculate about the production of DMHg in the deep ocean*”.

Reviewer #3 (Remarks to the Author):

Thank you for your thoughtful responses. I appreciate the clarification and found the majority of your edits in response to my comments to be sufficient. I have two queries below, the first of which, if I am interpreting everything correctly, would not require and additional modifications. The second is relevant to Reviewer 2’s critiques, which I think could be addressed further.

Original comment and response:
335-342: Why did you use fish tissues for the surface but particles for the intermediate depths? If you used fish values for both zones (or particles), would the weighted means come out the same?
No, the weighted means would be much higher if we use fish tissues, and much lower if we use particles for both zones, compared with currently estimated values. We think itis not theoretically correct to use fish tissues or particles for both zones to calculate the weighted means of upper ocean MMHg isotope compositions. The fish tissues from the surface ocean integrate the photodegraded MMHg isotope signatures in surface seawater, while the fish tissues from the intermediate ocean integrate the mixed isotope signatures between photodegraded surface MMHg and non-photodegraded intermediate MMHg, due to the downward MMHg transport from surface to intermediate oceans (see our Fig. 5 or Blum et al. 2013). The original isotope signatures of non-photodegraded in-situ MMHg in the intermediate seawater without mixture are theoretically imparted from those of particle Hg(II), because MMHg is thought to be microbially produced from particle-supplied Hg(II). This is why we use fish tissues from the surface ocean and particles from the intermediate ocean to calculate the weighted means of upper ocean MMHg isotope compositions.

Current comment:
The reasoning for using fish from the surface ocean makes sense to me, as you are only interested in MMHg and they are mostly MMHg. I just want to make sure that I am interpreting the use of particles for the intermediate ocean correctly. In theory, particles in the intermediate ocean would be bound to both IHg(II) and MMHg…some of the MMHg would have been produced in the upper ocean and have a photodegraded MMHg signature (relatively high MIF signature) and the rest would have a low MIF signature due to microbially mediated production at intermediate depths from IHg(II). In order to estimate this microbially produced MMHg MIF signature, you are using intermediate depth particles. Is the assumption that because %MMHg of Hg bound to particles is quite low, that the amount of photodegraded MMHg bound to sinking particles captured in the intermediate waters is not high enough to bias this estimate (what you are essentially measuring is the IHg(II) signature and using that as a proxy for intermediate water MMHg signature after correcting for the methylation shift)? If I am interpreting that correctly, using fish for surface and particles for intermediate makes sense.

The reviewer’s interpretation is correct, which is exactly the reason why we use surface fishes and intermediate particles to estimate the weighted means of upper ocean MMHg isotope compositions.

To make our points clearer, we have slightly revised (bold font) the relevant sentences in lines 331-334: In which, δ^202^Hg_surf_, Δ^199^Hg_surf_ and Δ^201^Hg_surf_ of MMHg in the surface ocean **(after photo-demethylation)** are represented by those of NPO fishes at 0-100 mbs (δ^202^Hg = 1.06 ± 0.35‰, Δ^199^Hg = 3.84 ± 1.15‰, Δ^201^Hg = 3.13 ± 0.97‰, 1SD), and δ^202^Hg_int_, Δ^199^Hg_int_ and Δ^201^Hg_int_ of MMHg in the intermediate ocean (**without photo-demethylation**) are represented by those of NPO marine particles (**mainly IHg(II)**) after correcting for -0.5‰ shift of δ^202^Hg during microbial methylation of IHg(II).

However, that is then relevant to Reviewer 2’s major critique, and my previous question as shown below:

Original comment and response:
106: other studies have seen relationships between %MMHg and isotope values. Any idea why you might not have seen it here? Is it just because the variation is small?
The reason for %MMHg vs. isotope values relationships seen in other studies is because their studied biota assimilate inorganic Hg(II) and MMHg from ambient environments which have quite different isotope values. The end-member mixture of varying proportions of inorganic Hg(II) and MMHg results in these relationships. However, in our study, these relationships are absent. This is because the biotic Hg(II) is derived from in-vivo demethylation of ingested MMHg by trench fauna rather than inorganic Hg(II) from ambient environments (falling particles or sediments or seawater). This in-vivo MMHg demethylation causes very limited isotope fractionation between MMHg and Hg(II). Thus, we observe very limited isotope variation in trench fauna in spite of large variation of MMHg%. We present four lines of evidence to support this view point, in our response to reviewer 2’s general comment #2 above. In the revised manuscript we have clarified the text to explain this better (see 2ndparagraph of Discussion).

Current comment:
Assuming the amphipods are consuming particles transporting both MMHg and IHg(II), but mostly IHg(II) as assumed above, it seems difficult to then assume that IHg(II) in the tissues is there solely from demethylation of ingested MMHg and they are not assimilating any IHg(II) from their food. The end member of ingested IHg(II) should somewhat similar to the intermediate water particle signature, if I am thinking this through correctly, and you assume no fractionation during trophic transfer. I agree with your arguments that demethylation is likely occurring, and that the MMHg is of surface origin, which would result in IHg(II) of similar signature for 199, with a slight shift of remaining MMHg for 202 if microbially demethylated, but…is there any way that particle bound IHg(II) transported to the deep water and ingested by the amphipods could have a similar signature to the MMHg…some combination of photochemical transformation and then ligand binding in the upper ocean (this would probably affect 202 most)? Or, mixing of particle signature and demethylated signature in vivo? Alternatively, is there any evidence you could present that excretion of ingested IHg(II) occurs at a greater rate than excretion of IHg(II) produced in vivo, which would result in retention and thus measurement of just the IHg(II) produced by demethylation (thus resulting in similar THg signatures across the range of %MMHg)? Is there evidence that bioavailable IHg(II) isotopic signature is closer to that of MMHg than to environmental IHg(II)? While you did add a reference regarding in vivo demethylation in marine mammals, I think it is important to demonstrate whether this has also been observed in crustaceans in other environments or not, if you are arguing that it is a critical process in driving observed isotope signatures. I don’t disagree with your interpretation, particularly as it relates to MMHg production in the upper ocean and transport to the hadal food web, but I wonder if there are other ways to explain why the IHg(II) and MMHg within the amphipod tissues have the same general signature (resulting in no relationship with %MMHg) that you could rule out and strengthen your arguments?

From the above comments, the reviewer wants to know if there are other ways (in addition to in-vivo demethylation that we propose here) to explain why IHg(II) and MMHg within the amphipod tissues have the same signature. More specifically, the reviewer wants to know: 1) is there any way that particle bound IHg(II) transported to the deep water and ingested by the amphipods could have a similar MIF signature to the MMHg, if IHg(II) undergoes photochemical transformation and then ligand binding in the upper ocean? 2) is it possible that IHg(II) in amphipods represents a mixing of particle signature and demethylated signature in vivo? 3) Is there evidence that bioavailable IHg(II) isotopic signature is closer to that of MMHg than to ambient, deep environmental IHg(II)? 4) is there any evidence you could present that excretion of ingested IHg(II) occurs at a greater rate than excretion of IHg(II) produced in vivo.

First of all, we thank the reviewer’s thoughtful queries. For #Q1-2, to our best knowledge, we believe the contribution from other sources of IHg(II) is very limited, because current field data show that IHg(II) in the open ocean, including particulate Hg, dissolved Hg, or deep sediment Hg, has very low D199Hg. It is possible that the photochemcial transformation of IHg(II) and ligand binding in the upper ocean could change its δ^20^2Hg and Δ^199^Hg, but the fractionation magnitude is supposed to be small, especially for Δ^199^Hg. We did not see significant variation in Δ^199^Hg of IHg(II) between upper and deep oceans. For Q#3, we are not aware of any reports that indicate that bioavailable IHg(II) has a very different D199Hg from bulk IHg(II), and would possibly have high Δ^199^Hg similar to our amphipods. For Q#4, we do have some evidences inferred from feeding strategies and physiological processes of amphipods. The trench amphipods inhabit an impoverished and harsh environment where food availability is lacking, and have evolved unique survival strategies. They typically have low metabolic rates and high lipid abundances for energy storage to cope with an extended period of starvation (Gerringer, 2019; Jamieson et al., 2010). This may facilitate the accumulation of lipophilic MMHg for a long period of time. The external IHg(II) fraction from food sources in amphipods is expected to be gradually excreted during starvation (most of our collected amphipods have little gut contents). Meanwhile, the gut microbiome in amphipods demethylates the accumulated MMHg and increases the in-vivo IHg(II) fraction. This effect appears to be more evident with the increase in life spans of amphipods, as seen from the negative correlation between MMHg% and body length (Fig. S2b), and the positive correlation between Δ^199^Hg and body length (Fig. S3).

In summary, although we cannot totally exclude the contribution of external food IHg(II) in amphipods, we demonstrate that this contribution is very limited, and unlikely changes the Hg isotope compositions of amphipods and compromise our interpretations. To address the review’s concern, we have slightly modified our text in 2^nd^ paragraph of Discussion (lines 148-171) to allow limited contribution from external food IHg(II).

Gerringer, M.E. (2019) On the success of the hadal snailfishes. Integ. Organ. Biol. 1, 1-18.

Jamieson, A.J., Fujii, T., Mayor, D.J., Solan, M. and Priede, I.G. (2010) Hadal trenches: the ecology of the deepest places on Earth. Trends Ecol. Evol. 25, 190-197.

**Reviewer comments, third version:**

Reviewer #3 (Remarks to the Author):

Thank you for your additional commentary; I am satisfied with your responses to my minor questions. This manuscript and the data interpretation has been much improved by your clarifications and comments throughout the review process.

**Author rebuttal, third version:**

Reviewer #3 (Remarks to the Author):

Thank you for your additional commentary; I am satisfied with your responses to my minor questions. This manuscript and the data interpretation has been much improved by your clarifications and comments throughout the review process.

We thank again the reviewer for the encouraging comments and previous comments.
